# Assessment of Trinidad community stakeholder perspectives on the use of yeast interfering RNA-baited ovitraps for biorational control of *Aedes* mosquitoes

**DOI:** 10.1371/journal.pone.0252997

**Published:** 2021-06-29

**Authors:** Nikhella Winter, Akilah T. M. Stewart, Jessica Igiede, Rachel M. Wiltshire, Limb K. Hapairai, Lester D. James, Azad Mohammed, David W. Severson, Molly Duman-Scheel

**Affiliations:** 1 Department of Life Sciences, The University of the West Indies, St. Augustine Campus, St. Augustine, Trinidad and Tobago; 2 Eck Institute for Global Health, University of Notre Dame, Notre Dame, Indiana, United States of America; 3 Department of Biological Sciences, University of Notre Dame, Notre Dame, Indiana, United States of America; 4 Department of Medical and Molecular Genetics, Indiana University School of Medicine, South Bend, Indiana, United States of America; Universidade Federal do Rio de Janeiro, BRAZIL

## Abstract

Dengue, Zika, chikungunya and yellow fever viruses continue to be a major public health burden. *Aedes* mosquitoes, the primary vectors responsible for transmitting these viral pathogens, continue to flourish due to local challenges in vector control management. Yeast interfering RNA-baited larval lethal ovitraps are being developed as a novel biorational control tool for *Aedes* mosquitoes. This intervention circumvents increasing issues with insecticide resistance and poses no known threat to non-target organisms. In an effort to create public awareness of this alternative vector control strategy, gain stakeholder feedback regarding product design and acceptance of the new intervention, and build capacity for its potential integration into existing mosquito control programs, this investigation pursued community stakeholder engagement activities, which were undertaken in Trinidad and Tobago. Three forms of assessment, including paper surveys, community forums, and household interviews, were used with the goal of evaluating local community stakeholders’ knowledge of mosquitoes, vector control practices, and perceptions of the new technology. These activities facilitated evaluation of the hypothesis that the ovitraps would be broadly accepted by community stakeholders as a means of biorational control for *Aedes* mosquitoes. A comparison of the types of stakeholder input communicated through use of the three assessment tools highlighted the utility and merit of using each tool for assessing new global health interventions. Most study participants reported a general willingness to purchase an ovitrap on condition that it would be affordable and safe for human health and the environment. Stakeholders provided valuable input on product design, distribution, and operation. A need for educational campaigns that provide a mechanism for educating stakeholders about vector ecology and management was highlighted. The results of the investigation, which are likely applicable to many other Caribbean nations and other countries with heavy arboviral disease burdens, were supportive of supplementation of existing vector control strategies through the use of the yeast RNAi-based ovitraps.

## Introduction

Mosquito-borne illnesses present a continuing threat to global health security. Despite implementation of disease management strategies, including preventive chemotherapy, vector control, and social mobilization, that seek to reduce the clinical burden, more than 3.5 billion people worldwide are still at risk of contracting vector borne diseases [1–3]. Suboptimal living conditions and polluted environments frequently overlap, creating circumstances in which medically important pathogens and their mosquito vectors thrive to enhance the rate of transmission and epidemic outbreaks. Although quantifying the burden of disease is challenging, it is estimated that one billion infections and one million deaths annually can be attributed to mosquito-borne diseases. While the mortality statistics are disturbing, they misinterpret the significance of morbidity that represents the human cost of these diseases in which permanent disability, lost productivity, and societal exclusion often contribute to desolation and poverty [4].

In recent decades, the incidence of arthropod-borne viruses (arboviruses) has increased dramatically due to multiple interconnected factors, including rapid urbanization and population growth. Collectively, these factors generate transmission settings conducive to the emergence [5–9] and re-emergence [10, 11] of mosquito-borne epidemic outbreaks [12]. Most global arbovirus infections are transmitted by two mosquito vectors, *Aedes aegypti* (L.) and *Aedes albopictus* (Skuse), which are geographically distributed throughout tropical and temperate regions, and vector chikungunya, dengue, yellow fever, and Zika viruses [13–15]. In the World Health Organization (WHO)-defined Americas region, which includes North, Central and South America plus the Caribbean, the number of infections recorded during the first half of 2020 exceeded 1.75 million (dengue: 1,731,786; chikungunya: 47,868; Zika: 8,277), with >600 deaths resulting from severe dengue [16]. The Republic of Trinidad and Tobago is a two-island state located at the southernmost tip of the Caribbean archipelago near the northeastern Venezuelan coast. Recent arboviral outbreaks in Trinidad and Tobago follow similar patterns to those observed across the WHO-Americas region. These outbreaks resulted from the establishment of *Ae*. *aegypti*, the primary arboviral vector, and *Ae*. *albopictus*, the secondary vector [17], as a result of the close association of these mosquitoes with densely populated urban environments in which rainwater-filled, artificial containers (e.g. tanks, drums, tires) provide breeding sites in which *Aedes* favor oviposition [18].

In the absence of licensed vaccines or chemotherapy, arboviral prevention and control is dependent on the effective management of *Aedes* populations in an effort to reduce the vectorial capacity of the mosquitoes below the threshold that sustains disease transmission. The Insect Vector Control Division (IVCD) of the Ministry of Health of Trinidad and Tobago (MoH), implements preventive approaches towards both juvenile and adult mosquito life cycle stages. Larval source management, through removal of standing water, as well as the application of biological (*Bacillus thuringiensis israelensis* (*Bti*)) and chemical (Aquatain™) larvicides, is a key element of control efforts in Trinidad and Tobago, where adults are also targeted by ultra-low volume (ULV) fogging with the organophosphate insecticide, malathion. Although these approaches continue to be the mainstay of mosquito control on the islands, the effectiveness of insecticide-based approaches may be severely threatened by the establishment of resistance [19, 20] to the major insecticide classes approved for public health use by the WHO [21]. Health and safety concerns surrounding toxicity and its ecological impact on non-target organisms are additional factors that highlight a critical need for the development of alternative vector control methodologies which attempt to address these limitations. To this end, researchers are exploring the use of new mosquito control technologies that could be useful additions to integrated mosquito control programs. The current investigation explores the potential use of RNAi interference (RNAi), as a new species-specific method of mosquito control.

RNAi is an evolutionarily conserved mechanism that regulates gene expression through the production of small noncoding RNAs. Initiated by the presence of interfering RNA (iRNA) species, the RNAi pathway is a cellular cascade of sequence-specific molecular interactions that results in translational repression or degradation of target mRNA transcripts to effectively silence gene expression. Although the United States Environmental Protection Agency (EPA) has registered an RNAi-based strategy that targets an agricultural pest [22, 23], the application of this technology for the control of medically important mosquito disease vectors in the field is unexplored [24]. High-throughput screens [25, 26] identified hundreds of small interfering RNA (siRNA) pesticides that kill mosquito larvae. A subset of the larval lethal siRNAs target multiple species of mosquitoes that vector major diseases, including *Ae*. *aegypti* and *Ae*. *albopictus*, through shared sequence homology [27–30]. No other known organism returns an *in silico* match to these siRNAs, emphasizing their sequence-specificity and potential as a biorational larvicide application with minimal non-target impact. Extensive use of this technology, however, requires an effective, economic, and user-accepted system of integrating RNAi larvicides into operational mosquito control programs.

Oral delivery of iRNA, which is often used for gene silencing in the laboratory, is being assessed as a potential delivery mechanism for larvicidal control operations in the field [24]. Recently, *Saccharomyces cerevisiae* yeast has been identified as a promising iRNA expression and delivery system. Numerous strains, each expressing a short hairpin RNA (shRNA) [31] that targets larval lethal genes identified in the screens, have been generated [25, 26]. In addition to genetic tractability, *S*. *cerevisiae* has several physical properties that are of benefit to an operational mosquito control program. As a strong odorant, it can be used in a “lure-and-kill” approach to attract gravid females to treated aquatic breeding sites [25]. It also serves as a nutritional source to developing larvae and can be easily cultured, heat-inactivated, and prepared as dried tableted [31] formulations, which can simply be added to breeding sites for oral ingestion. A yeast delivery system also represents an affordable method of synthetic RNA propagation since commercial fermentation processes are available [32]. Importantly, larvicidal activity is not affected by heat-inactivation of *S*. *cerevisiae*, which is applied in the field as a dead microbe rather than as a live GMO, a salient point that could influence field operations and public acceptance of the new mosquito control intervention.

Recent laboratory and semi-field studies have demonstrated the efficacy of yeast iRNA larvicides [25–30], and the potential for scaled production of commercial formulations [32] is currently being explored. Successful integration of this technology into disease-endemic countries requires effective community engagement prior to, during, and following the deployment of new mosquito control interventions [33, 34]. The theoretical basis for effective community engagement in global health research is well-described by Lavery et al. [35], a publication which considered concepts from sociology, anthropology, political science, community development, agriculture, environmental and public health, civil society and non-academic literature, to develop a list of considerations for effectual community engagement in global health studies. The publication emphasized the early initiation of activities that allow investigators to communicate the purpose and goals of the research program and establish relationships and commitments to build trust with stakeholders [35], defined herein as members of a community that can affect or be affected by mosquitoes and mosquito control measures implemented in the areas in which they reside. Such activities also provide an opportunity for the researchers to understand the community, its diversity, and evolving needs, and to maximize opportunities for stewardship and shared ownership and control of the new intervention. Importantly, these interactions also create a platform for the expression of dissenting opinions, providing researchers the opportunity to amend the proposed studies, or in extreme cases, to delay or end the research program [35]. Lavery et al. [35] also emphasized that effective community engagement should extend beyond surveys and include activities that allow community members to express their perspectives in their own terms rather than solely relying on concepts and pre-determined survey questions prepared by the investigators. This point shaped the current investigation, which emphasized community engagement activities that extended beyond initial paper surveys and promoted direct interactions between the researchers and community members through forums and interviews.

A recent study in Trinidad concluded that the participants are supportive of the potential use of yeast interfering RNA larvicides in Trinidad [34]. The study, which identified several prospective field sites to test this new technology, focused on the use of the larvicides to treat artificial container habitats (i.e. barrels, basins, tubs, and other water storage containers). The present study further engaged Trinidadian stakeholders, who were introduced to another application of the RNAi yeast technology: the development and use of iRNA yeast-baited ovitraps. Lure-and-kill yeast iRNA ovitraps, water-filled containers treated with iRNA yeast larvicides that are designed to lure gravid mosquitoes to lay eggs in containers in which the resulting larvae will be killed, are presently being evaluated and optimized in Trinidad. Although ovitraps are often used as a means of mosquito surveillance, the ability to install the ovitraps at a high enough density to permit mosquito control, through reduction of larval offspring that die upon hatching in the insecticide-treated ovitrap containers, is the subject of an ongoing investigation that is being conducted in Trinidad. In the present study, it was hypothesized that ovitraps would be broadly accepted by community stakeholders as a means of biorational control for *Aedes* mosquitoes. To examine this hypothesis, three types of assessment tools—paper surveys, community engagement forums, and household interviews—were implemented to assess feedback from a subset of community-stakeholders across Trinidad (S1 Fig). Paper survey studies and community engagement forums preceded field studies in which the traps were placed at residential properties, giving the residents an opportunity to provide experiential feedback. These studies were pursued in an effort to create public awareness of this new vector control strategy, to gain stakeholder feedback regarding product design and acceptance of the new intervention, and to build capacity for its potential integration into existing mosquito control programs. These assessments, which facilitated analysis of local community stakeholders’ knowledge of mosquitoes, vector control practices, and perceptions of the new technology, permitted evaluation of the hypothesis that the ovitraps would be broadly accepted by community stakeholders as a means of biorational control of *Aedes* mosquitoes. Results, together with a comparison between survey instruments, and analysis of the merits of each assessment method in this setting are reported. The results of the investigation, which are likely applicable to other Caribbean Island nations and potentially to other countries with heavy arboviral disease burdens, were supportive of the new technology.

## Materials and methods

### Ethics statement

The human subjects protocol used in this investigation was approved by the Indiana University (IU) Human Subjects Office (protocol 1608074907A008), the University of Notre Dame (ND) Office of Research Compliance (Protocol #17-07-3984), the Trinidad and Tobago South West Regional Health Authority Ethics Committee, the University of the West Indies at St. Augustine, Trinidad and Tobago (UWI) Ethics Committee (Study CEC403/12/17), and the U.S. Army Medical Research and Materiel Command, Office of Research Protections (ORP), Human Research Protection Office (HRPO) (log numbers A-20339.1a, A-20340, and A-20339). The research was deemed exempt, and therefore no consent was required, as participation in the study was not deemed to present any risk to participants.

### Paper survey

**Fig 1:**
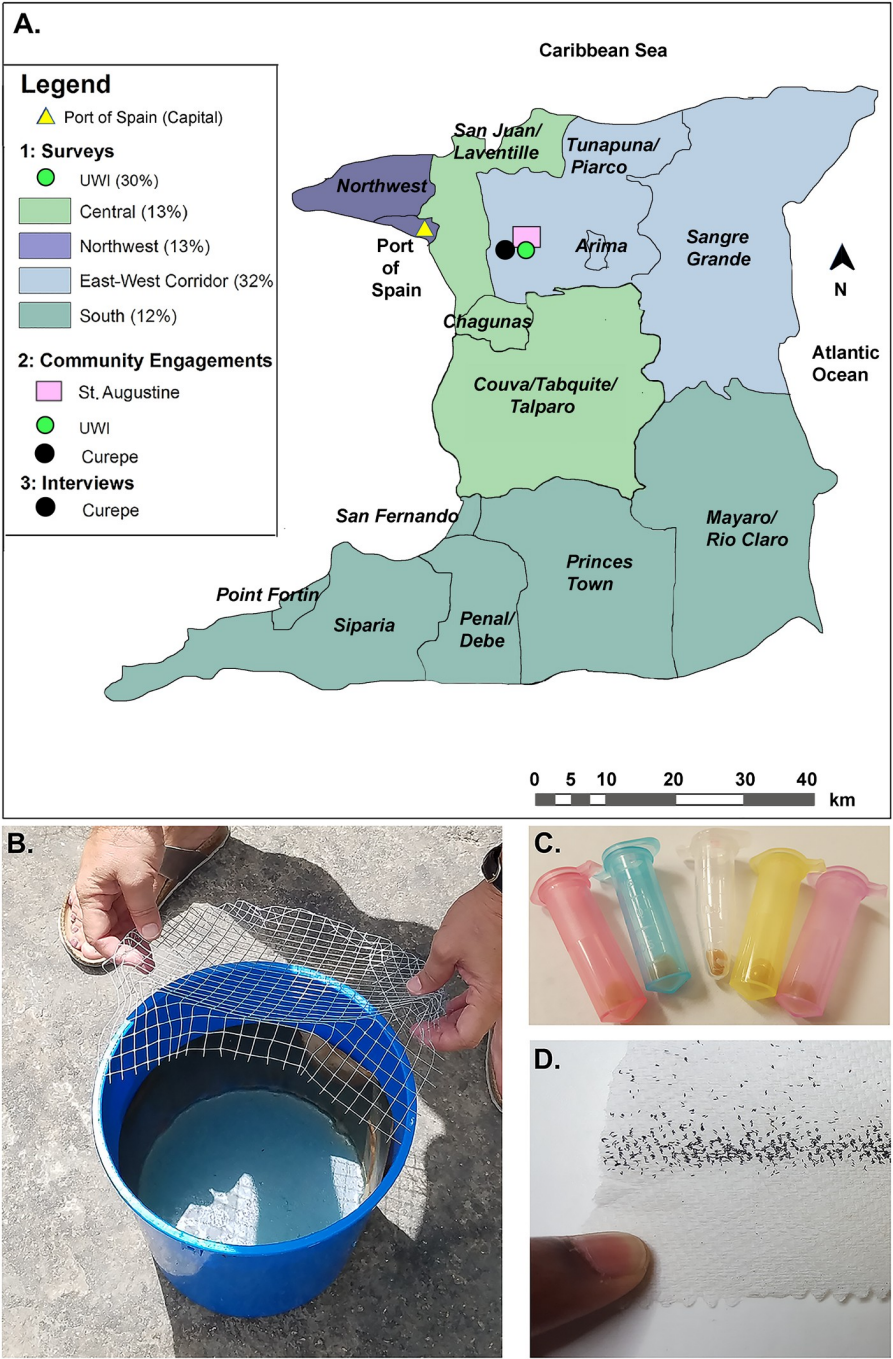
Study site data collection areas and props. A. Locations of the paper survey sampling regions with percentages of samples collected in the Central, East-West Corridor, Northwest, and South regions as well as the UWI St. Augustine Campus are shown. Community engagement forums were conducted at UWI and St. Augustine, and household interviews were conducted in the adjacent community of Curepe. The map image was generated using ArcMap v.10.3.1 [37]. Larval-lethal ovitraps (B), yeast samples (C), and *A*. *aegypti* eggs (D) were used as props in the community engagement forums.

Paper survey study information sheets (S1 File) and surveys (S2 File) were administered in a manner similar to the approach in Stewart et al. (2020) [34] during the 12-month period from March 2018–2019. The paper survey instrument (S2 File) included twelve 5-point Likert-scale statements, which were designed to examine local participants’ general knowledge, current practices, and attitudes toward mosquitoes and their control. For the design of questions included in this assessment tool, as well as the engagement forum and interview questions described below, questions were written on the basis of the scientific aims of the study, following a general review of the vector literature on this topic, and through refinement of the questions by the research team, MoH staff, and by non-scientist community stakeholders. An optional demographic response section was included in each survey. Survey collection locations were grouped into four broad regions based on Trinidad’s thirteen established administrative Regional Corporations, and a fifth collection area, the UWI St. Augustine Campus (Fig 1). These regions spanned: Central (Chaguanas and Couva-Tabaquite-Talparo Regional Corporations), Northwest (Diego Martin and Port of Spain Regional Corporations), East-West-Corridor (Tunapuna-Piarco, San Juan-Laventille and Sangre Grande Regional Corporations), and South (Penal-Debe, Princes Town and Siparia Regional Corporations), with roughly the same number of individuals from each region included in the study. Individuals who participated in the community engagement forums (see more details below) were also included in this study group. In such cases, participants were invited to complete the surveys prior to forum participation, to circumvent skewed responses based on new information learnt during these events. To target a wide variety of participants, surveys were distributed in crowded places such as shopping malls and schools. It was determined a priori, based on a general survey of publications that described paper survey data, as well as through the use of similar assessment tools in Trinidad in a related study [34], that 500 people would be targeted, and this goal was achieved (n = 500). Demographics of the study participants were determined to be representative of the population of Trinidad prior to the conclusion of survey distributions. Survey responses were evaluated statistically with Qualtrics StatsIQ [36]. Responses from all respondents were included in these analyses, even when a respondent elected to skip an individual survey question, which occurred occasionally and is reflected in the quantitative results reported below, which were acquired from the Qualtrics StatsIQ software.

### Community engagement forums

Community engagement forums were held at four locations near to the prospective field trial site of Curepe, with this potential trial site chosen on the basis of proximity to the research laboratory, relative ease of travel to the study site, the density of mosquitoes in the area, and following completion of a related study which concluded that stakeholders in the area were generally supportive of the potential use of yeast interfering RNA larvicides [34]. Engagement forum locations included the UWI campus, St. Augustine Community Centre, a residential site on Old Tim Road, and the St. Augustine Secondary School. Events were advertised through electronic notices, posters, public announcements using a car equipped with a loudspeaker (culturally relevant means), and by inviting pedestrians in the vicinity thirty minutes before the event commenced. Refreshments were made available during and/or after the events and functioned to encourage both attendance and participation, which varied based on the density of people living in the study site regions. Every effort was made to recruit broadly in the areas in which the events were held, with the timing of each event (late afternoon/early evening) selected on the basis of the relative availability of most participants at this time of day. Although no data allowing for the identification of any specific participant were collected, participants were screened to ensure they were Trinidad and Tobago residents aged 18 years or older. Participants were provided a study information sheet (S3 File) prior to the start of the forum and were encouraged to fill out demographic information sheets (S4 File).

Each event was led by a native Trinidadian member of the UWI research team and followed a similar program. First, the moderator introduced herself and other members of the combined UWI, IU, and ND team in attendance, then proceeded to give a short summary of the project, which included a brief demonstration of ovitrap use and product application. Participants were presented with general information relating to mosquito ecology and the competence of *Aedes* as a vector, as well as contrasting methods of vector control including conventional techniques and yeast iRNA-baited ovitrap technology. This was in the form of a prepared introductory script (S5 File) so that it was standardized across each engagement and included the use of ovitraps (Fig 1B), yeast tablets (Fig 1C), and *A*. *aegypti* eggs (Fig 1D) as props. Once participants became more familiar with the study content, a guided group discussion commenced using a series of scripted questions (S6 File). This discourse revealed respondents’ feelings about the technology and assessed their levels of acceptance. The sessions concluded with experts from IU and ND responding to scientific questions that arose during the dialogue. Each of the four sessions were audio recorded and used by native Trinidadian UWI researchers to generate transcripts for further analysis.

### Household interviews

#### Study site

To assess acceptability of the proposed technology, a random sample of face-to-face household interviews were conducted over a six-month period from July 2019 to December 2019. The interviews were conducted in parallel to a yeast-baited ovitrap field trial that was performed in collaboration with the Insect Vector Control Division (IVCD) of the MoH. Interviews, as well as the ovitrap field study, were conducted with consenting households in parallel to IVCD routine surveillance visits to these households, during which time IVCD staff gained permission from residents for both staff and the research team members to access the residences. The study site consisted of nine discrete blocks distributed in Curepe (range: ~10,000–25,000 m^2^), which were comprised of a mixture of private residential homes, rental apartments, and small business buildings. From these blocks, 94 properties were identified as suitable to position ovitraps, and 29 interviewees were selected from 23 of these households on the basis of availability and willingness to participate. Interviews were intentionally completed during times when individuals living in the homes were available.

#### Interview survey instrument

Household interviews aimed to document an individual’s feelings about the approach in general, its utility, and its demonstrated practicality. Participants were provided with a study information sheet (S7 File) and an optional demographic sheet (S8 File). Interviews were conducted by UWI researchers and followed a series of eight scripted questions (S9 File). Questions were designed to assess participants’ experiences with having the larvicidal ovitraps on their properties, impression of its applicability, and willingness to integrate the approach habitually. Interviews were conducted during ovitrap servicing periods (09:00–13:00 hrs), and responses were audio recorded by consent. Each recording lasted 10 minutes/individual on average.

#### Transcript analysis

Transcripts were generated by UWI researchers using audio recordings from community engagement forums and interviews, then combined into single documents, respectively, as per Stewart et al. [34]. Analysis of sentences and quotes led to general groups termed codes, as well as more specific categories. Text analysis was implemented with Text Analyzer [38], permitting identification of keywords or significant phrases that were assessed with greater resolution.

## Results

### Paper surveys

#### Participant demographics

**Fig 2:**
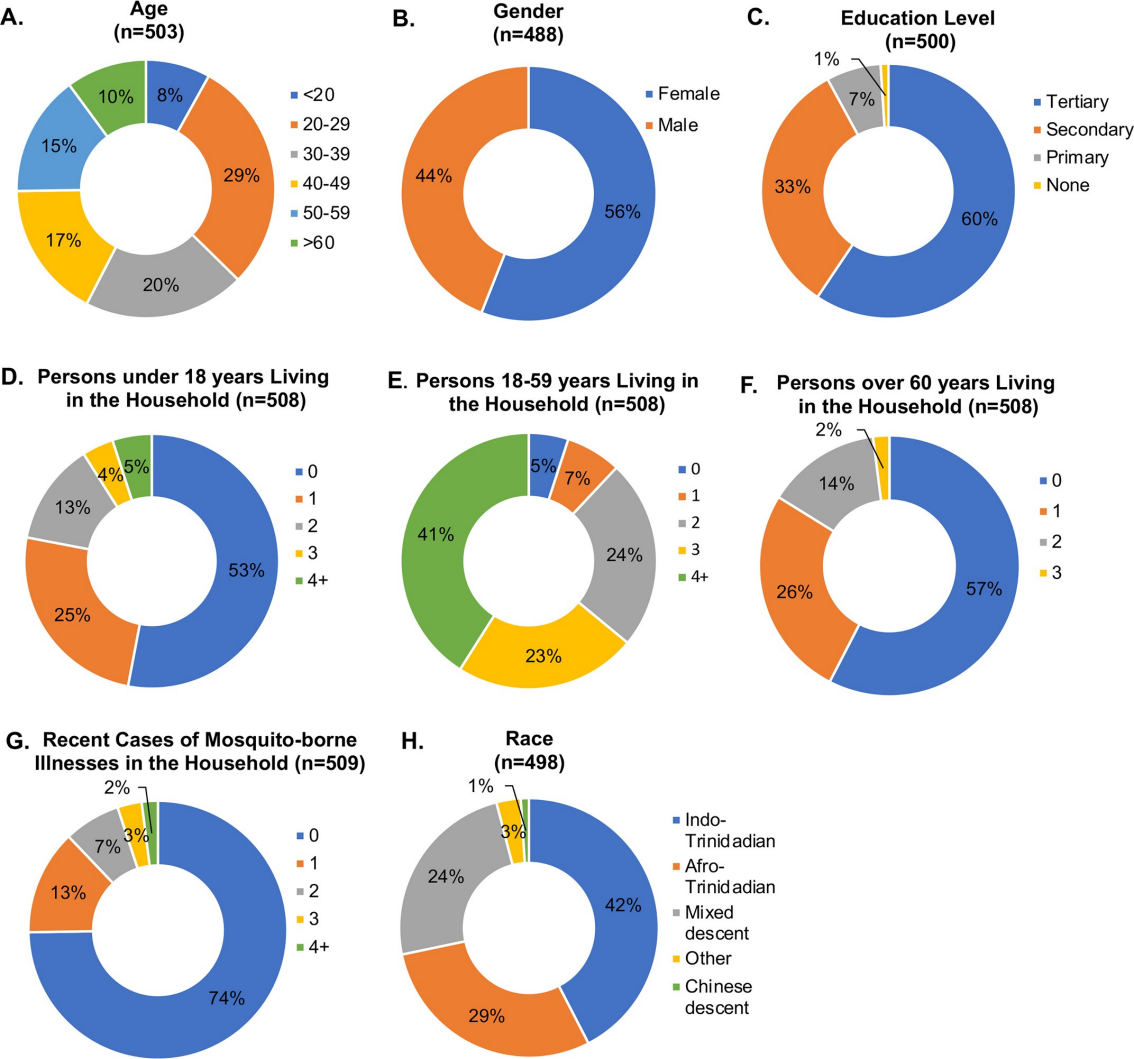
Demographic information for survey respondents. Data on participants’ age, gender, race, highest level of education attained or enrolled, the age of persons in the participant’s household, recent cases of mosquito-borne illness in the household, and race are summarized.

A total of 513 survey responses were collected from the Central, East-West Corridor, Northwest, South, and UWI regions (S1 Table). Demographic data for the respondents (Fig 2) are indicative of a diverse group of participants who varied in age (Fig 2A), gender (Fig 2B), formal education level (Fig 2C), and race (Fig 2H), generally reflecting data gathered in the 2011 census [39], except that gender distribution (Fig 2B) demonstrated a greater number of female participants (274 of 488, 56.1%) than males (214 of 488, 43.9%), and a greater ratio of participants had attained or were enrolled in secondary-level education (463 of 500, 92.6%, Fig 2C). A large proportion of respondents were aged 20–29 years (148 of 503, 29.4%) followed by 30–39 years (101 of 503, 20.1%; Fig 2A). Indo- (208 of 498, 41.8%) and Afro-Trinidadian (145 of 498, 29.1%) were the most frequently-listed races followed by mixed descent (120 of 498, 24.1%), other (16 of 498, 3.2%) and Chinese descent (7 of 498, 1.4%) (Fig 2H). Households often included both children under age 18 and adults over age 60 (Fig 2D–2F). 258 of 509 respondents (25.7%) indicated that someone in their household had experienced dengue, Zika, chikungunya, and/or yellow fever one or more times during the past two years (Fig 2G).

#### Responses to survey questions

**Fig 3:**
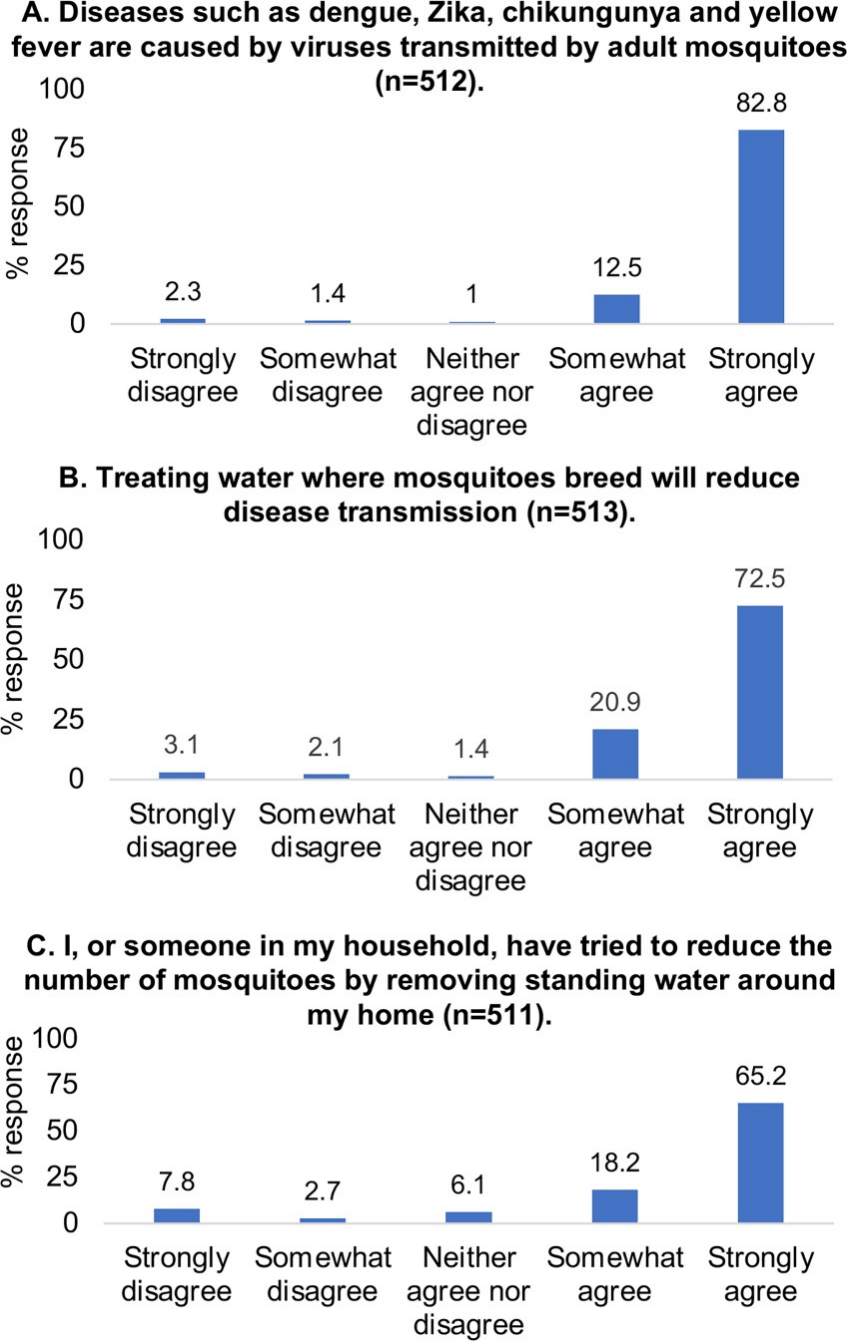
Paper survey responses to general mosquito knowledge questions. A summary of responses to the first three paper survey questions, all of which assessed general mosquito knowledge, is presented.

**Fig 4:**
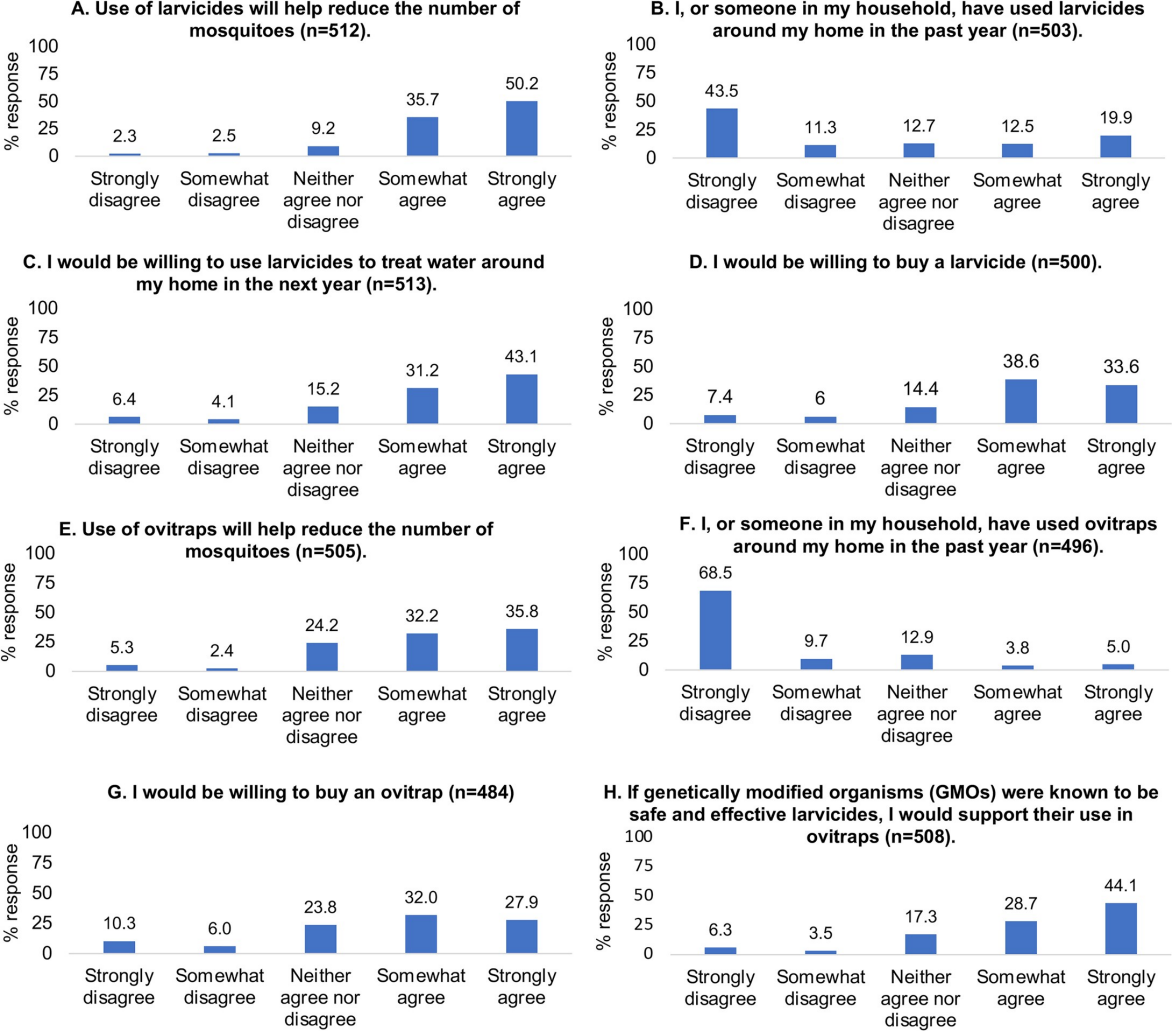
Paper survey respondents’ mosquito control practices and perceptions. A summary of responses to eight questions pertaining to larvicides and ovitraps is presented.

When participants’ knowledge of mosquito-borne illnesses and control practices were assessed, 488 of 512 (95.3%) agreed that dengue, Zika, chikungunya, and yellow fever were viruses transmitted by adult mosquitoes (Fig 3A). 479 of 513 (93.4%) respondents demonstrated some understanding of disease transmission by agreeing that treating aquatic sites where mosquitoes breed reduces transmission (Fig 3B). 440 of 512 (85.9%) individuals also agreed that the use of larvicides would help to reduce the number of mosquitoes (Fig 4A). When prompted for a response to the same statement, but with ovitraps as the control intervention, there was a decrease in the number of respondents (344 of 505, 68.1%) who agreed that ovitrap use would reduce the number of mosquitoes (Fig 4E). Interestingly, this decrease was accompanied by an increase in “Neither agree nor disagree” responses from participants (122 of 505, 24.1%) compared to previously answered statements.

426 of 511 (83.4%) respondents agreed that they, or someone in their household, had attempted to reduce the number of mosquitoes by removing standing water (Fig 3C). This contrasted sharply with the use of chemical control interventions, with only 163 of 503 (32.4%) individuals agreeing to have used larvicides (Fig 4B) and 44 of 496 (8.9%) agreeing to having used ovitraps during the past year (Fig 4F). Generally, most respondents were willing to adopt existing or alternative interventions. 381 of 513 (74.3%) respondents were willing to use larvicides to treat water around their home in the upcoming year (Fig 4C), and a similar number (361 of 500, 72.2%) were willing to purchase the larvicides (Fig 4D). A majority of respondents (290 of 484, 59.9%) were willing to purchase ovitraps (Fig 4G). When prompted to suggest a price they would be willing to pay for each mosquito control intervention, respondents suggested a range of prices from TT$1–1000 (US$0.15–148.00). The mean suggested price for a larvicide was TT$65.90 [Confidence interval (CI): 58.0–73.8] and for an ovitrap was TT$69.27 (CI: 59.7–78.8). Support for the use of GMOs in ovitraps was mostly positive (370 of 508, 72.8%) with only 50 of 508 individuals (9.8%) recording a negative response (Fig 4H).

Those who had tried to reduce the number of mosquitoes by removing standing water around their homes were more likely to strongly agree that treating water reduces disease transmission (258 of 511, 50.5%, P < 0.001, χ^2^ = 37.8, *df* = 16). Similarly, those who strongly agreed that they or someone in the household had used larvicides in the past year were more likely to strongly agree that use of larvicides reduces the number of mosquitoes (72 of 502, 14.3%, P < 0.001, χ^2^ = 51.1, *df* = 16). Those who strongly agreed that they or someone in the household had used ovitraps in their home in the past year were more likely to strongly agree that use of ovitraps would help reduce the number of mosquitoes (18 of 490, 3.7%, P < 0.001, χ^2^ = 40.4, *df* = 16). An understanding of control tools and practices appeared to influence purchasing decisions. Those who strongly agreed that treating water where mosquitos breed would reduce disease transmission tended to strongly agree that they would be willing to buy a larvicide (136 of 500, 27.2%, P = 0.008, χ^2^ = 32.7, *df* = 16), while those who strongly agreed that treating water where mosquitos breed would reduce disease transmission tended to strongly agree that they would be willing to buy an ovitrap (107 of 484, 22.1%, P = 0.022, χ^2^ = 29.3, *df* = 16). Practical experience and familiarity with a control method also tended to correlate with an individual’s willingness to purchase control products in the future. Those that tended to strongly agree that they would be willing to buy a larvicide tended to strongly agree that they or someone in their household had used larvicides in the past year (66 of 491, 13.4%, P < 0.001, χ^2^ = 90.4, *df* = 16). Similarly, those who strongly agreed that they would be willing to buy an ovitrap tended to strongly agree that they or someone in the household had used ovitraps in the past year (18 of 475, 3.8%, P < 0.001, χ^2^ = 46.2, *df* = 16).

### Community engagement forums

#### Participant demographics

Details relating to the dates of the events at the respective locations and number of participants in attendance at each forum are summarized in S2 Table. A total of 113 individuals participated in community engagement forums and 86 of 113 (76.1%) participants provided demographic information, which is summarized in S2 Fig. These demographic data generally agreed with that of the paper survey respondents in that most were represented by the 20–29 years age group (31.4%), and a greater number of females (51.8%) than males (48.2%) confirmed their gender. 96.5% of individuals had attained, or were enrolled, in secondary-level education. Indo-Trinidadian ethnicity (43.0%) aligned with paper survey and census data [39] but less Afro-Trinidadian individuals (22.1%) were represented. Although attendees were not specifically queried regarding their professions, discussions that evolved during the engagement events revealed that the attendees included individuals of a variety of professions, including teachers, students, scientists, an engineer, agricultural workers, secretaries, a landscaper, housewives, parents with children, and retirees.

*Summary of responses to scripted community forum questions*. A summary of the participants’ responses to the six survey questions (S6 File) is provided below.

***Question 1*. *What is your impression of how well the larvicidal ovitraps we have developed may work to control mosquitoes on your property*? *What do you think of this approach*?** From the four community engagements, 19 of 113 (16.8%) of the participants responded to this first question. Feedback included positive interest in the approach based on its safety and assumed simplicity such as “I like the idea that he [scientific expert] said that the yeast attracts the mosquitoes and the bucket [ovitrap] attracts the mosquitoes”. The question elicited concerns related to the targeted mosquito species as well as inquiries about the yeast iRNA larvicide product, the inclusion of the product in the ovitrap system, and the overall operation of the trap. Participants were particularly engaged by topics that dealt with mosquito biology and its influence on product efficacy. They questioned the lifespan of the mosquito and its life cycle behavior including the number of eggs a single female is able to lay.

***Question 2*. *Is there anything about the larvicidal ovitraps we described that you particularly like*?** There were 45 points of interest reported for this question, with some respondents providing multiple comments. 21 of 45 (46.7%) responses highlighted product safety for human health and the environment as preferential in comparison to aerosolized chemical-based product alternatives. 11 of 45 (24.4%) responses indicated that the respondents were pleased by some aspect of the larvicidal ovitrap, such as viewing it as a good idea or liking the approach to mosquito control. 6 of 45 (13.3%) respondents expressed a view that it was easy to use. 6.7% of responses (3/45) disclosed the respondents’ appreciation for their own ability to utilize the ovitrap system for mosquito management at a personal property level, thereby removing total dependency on MoH-IVCD interventions. This question also solicited more inquiries from the attendees. These included questions about operation “How effective would this trap be seeing as the drain is a competitor?” and “I would like to know…if it is possible to get more than the recommended number amount [of ovitraps]?”

***Question 3*. *Is there anything about the larvicidal ovitraps we described that you did not like*?** Only 3 of 113 (2.7%) respondents reported disliking some aspect of the yeast product. One participant perceived the ovitrap system to be high maintenance, indicating that it may become an effort to clean.

***Question 4*. *When you think about choosing among product options for mosquito control on your property*, *which factors are most important to you*?** Participant responses to this question are shown in Table 1. Safety ranked first, with 63 participants agreeing that it was the most important factor to consider when choosing a control option. Eco-friendliness ranked second, and other factors such as smell, effectiveness, cost, and access were also mentioned.

**Table 1:** Factors that determine mosquito control product selection.

Response	Count
Safety	63
Environmentally-friendly	58
Smell	33
Effective	31
Cost	27
Accessibility	24
Ease of use	8
Aesthetics	3

Counts signify the combined number of participants from the four community engagement forums with the indicated response.

***Question 5*. *If the larvicidal ovitraps we described were available for purchase*, *would you be interested in buying them*? *If so*, *what do you think a reasonable price for a monthly supply would be*?** 56 of 113 (50.0%) attendees indicated that they would be interested in buying the larvicidal ovitraps. Participants did not express any genuine disinterest in purchasing the larvicidal ovitraps. Only one person expressed that interest was contingent on the success rate. 11 of 113 (9.7%) participants suggested prices. TT$68.18 (CI: $32.20-$104.20) was the mean price recommended among the participants (range: TT$10-$210). There were two requests that product samples be distributed prior to purchase.

***Question 6*. *Is there anything else you would like to tell us about the larvicidal ovitraps we are testing*?** Additional questions that sought to gain more information arose. Some related to operation, such as “How many you need for proper coverage” or product design, such as “Could you leave some for us to try out to see how effective it is?” There were also questions relating to mosquito ecology, particularly lifespan, oviparous cycle and egg production. The degree of involvement by IVCD personnel was queried twice: once in support of their inclusion in the study and another in opposition to their involvement. The latter suggestion was agreed to by 23 of 113 (20.4%) other participants. Other questions/suggestions about commercialization and product development were also noted. More detailed analyses are provided in the sections that follow.

#### Transcript analysis for community engagement forums

Transcripts generated from public community engagement audio recordings were coded into six general categories, as follows: 1) information gathering questions, 2) positive comments, 3) negative comments, 4) neutral comments, 5) knowledge (the communication of existing stakeholder knowledge relevant to the discussion), and 6) recommendations for product design and optimization (Table 2). The largest coded category at these events was recommendations with 255 of 639 (39.9%) responses, followed by information gathering questions with 176 of 639 (27.5%) responses, and positive statements with 148 of 639 (23.2%) responses. 51 of 639 (8.0%) comments involved knowledge sharing. Few comments were negative (6 of 639, 0.9%) or neutral (3 of 639, 0.5%) comments.

**Table 2:** Analysis of the community engagement forum transcript data.

Code	Representative Quotes	Number of Comments	% of Total Comments
**Information gathering**	• What if what is trapped there… is there a way you could add something from this system to those plants to put these in your garden to prevent mosquitoes from breeding there?	176	27.5
	• How long do you leave the yeast in the bucket before changing it?		
	• Or is more traps you have the more you attract the mosquitoes?		
	• How does your product compare with the one that the Ministry has of marking houses?		
**Positive**	• I like that you tended to use that and combined with the scent of the yeast, would attract them [reference to bucket color].	148	23.2
	• Well, I particularly like that it is designed to target just the mosquitoes and not any other insects.		
	• I think it’s a really good cost-effective methodology that you all approached.		
	• With your traps, as you all have stated, will also help the environment, and would keep the environment clean and you wouldn’t have all those different chemicals.		
**Neutral**	• Even though I don’t care about the appearance.	3	0.5
	• Well, I guess.		
**Negative**	• I think it’s going to be a hassle to clean.	6	0.9
	• It is really appealing.		
**Knowledge**	• I really think we have some mutant mosquitoes around and I think it is simply because we are interfering with the natural order of things.	51	8.0
	• I live in a dark area and mosquitoes are attracted to damp things.		
	• Our mosquitoes usually breed in stagnant water.		
	• Some of the most common methods that we have cover their container.		
	• The bad thing is that it’s outside range so that we are not really fixing the problem, we are just protecting ourselves.		
	• You have to make sure it working for everything because although it works for mosquitoes, if you go on the field, bees attracted to it, and other insects.		
**Recommendations**	• If it has to be marketing, it has to be self-cleaning…biodegradable.	255	39.9
	• I think that our government should get involved.		
	Say we just have to put the yeast pellet, let’s say, once for a month, that would be awesome versus once every three days.		

The type, number, percentage of total, and representative quotes for each code are shown.

A detailed review of the information gathering questions revealed thirteen main themes (Table 3). Comments concerning the yeast iRNA-baited ovitrap approach were most common, representing 23 of 147 (15.6%) questions, including those particularly related to better understanding the concept. These were followed by questions related to the research on the yeast (18 of 147, 12.2%), such as duration of potency and local availability. In keeping with the theme of safety, there were many questions relating to the impact of yeast iRNA larvicide use on human health and the environment (18 of 147, 12.2%), including clarification of the mode of action and any potential effect on drinking water. Further questions concerned life history of the target species (17 of 147, 11.6%).

**Table 3:** Common categories of information gathering questions at the community engagement forums.

Category	Count	Representative Quote
Yeast-baited ovitrap approach	23	Just to reiterate, it’s the yeast that attracts them to these buckets, yeah?
Further information about yeast/Research	18	Is it that it’s normal yeast we use at home that could be poured in a bucket or in a small drain?
Human and environmental safety	18	What is the effect towards the environment?
Mosquito life history traits	17	What is the lifespan of the *Aedes*?
Price	12	…costly. Would it be like for twice a year or if it’s something that is consumable?
Application procedure and operations	11	How to do you use? You just take it and put it into a bucket?
Frequency and dose of treatment	10	What is the percentage of those who might be killed?
Commercialization	8	Are you all including the trap with the yeast?
Target group	8	Would it kill other mosquitoes or only the *Aedes aegypti*?
Availability	6	And how long people have to wait before you all product comes out?
Residual activity	5	When you insert one of those pellets, how long would the potency last?
Product design	5	What attachments do you have for the bucket so that it stays propped up?
Mosquito Control	3	The Ministry of Health has field officers who would go around from house-to-house, right, and taking samples?

Counts (of 147 total questions) and representative quotes for each theme are presented.

Many respondents offered advice with respect to the product and its design (Table 4). Recommendations were dominated by safety-related comments (75 of 228, 32.9%) followed by cost and suggestions (48 of 228, 21.1%). These included the potential for having the Trinidad and Tobago MoH or global health organizations contribute to the purchase, distribution, or maintenance of the ovitraps. Product design (13 of 228, 5.7%) recommendations included discussion of other domestic containers that might be converted into ovitraps. Further, suggestions to promote ease of operation (12 of 228, 5.3%) and use (such as the addition of a handle to the traps), larvicide formulation longevity (10 of 228, 4.4%), and the specificity of insects targeted (9 of 228, 4.0%).

**Table 4:** Categories of recommendations offered by attendees of the community engagement forums.

Category of recommendations	Count	Representative Quote
Safety for Humans/Environment	75	I a little concerned about the food chain along the line.
Cost/Pricing	48	I’d pay $50 for the bucket but $20 to refill it.
Efficacy	33	Effectiveness.
Availability	28	I think that our government should get involved.
Product design	13	Aesthetics/appearance.
Ease of operation	12	I could spend two minutes, every three to six months, to clean a bucket that completely eradicates mosquitoes from my home.
Formula longevity	10	I’d say once every two weeks or once a month.
Specificity	9	You have to make sure it’s specific.

A selection of the 228 recommendation quotes representing each category from across the engagement forums.

**Fig 5:**
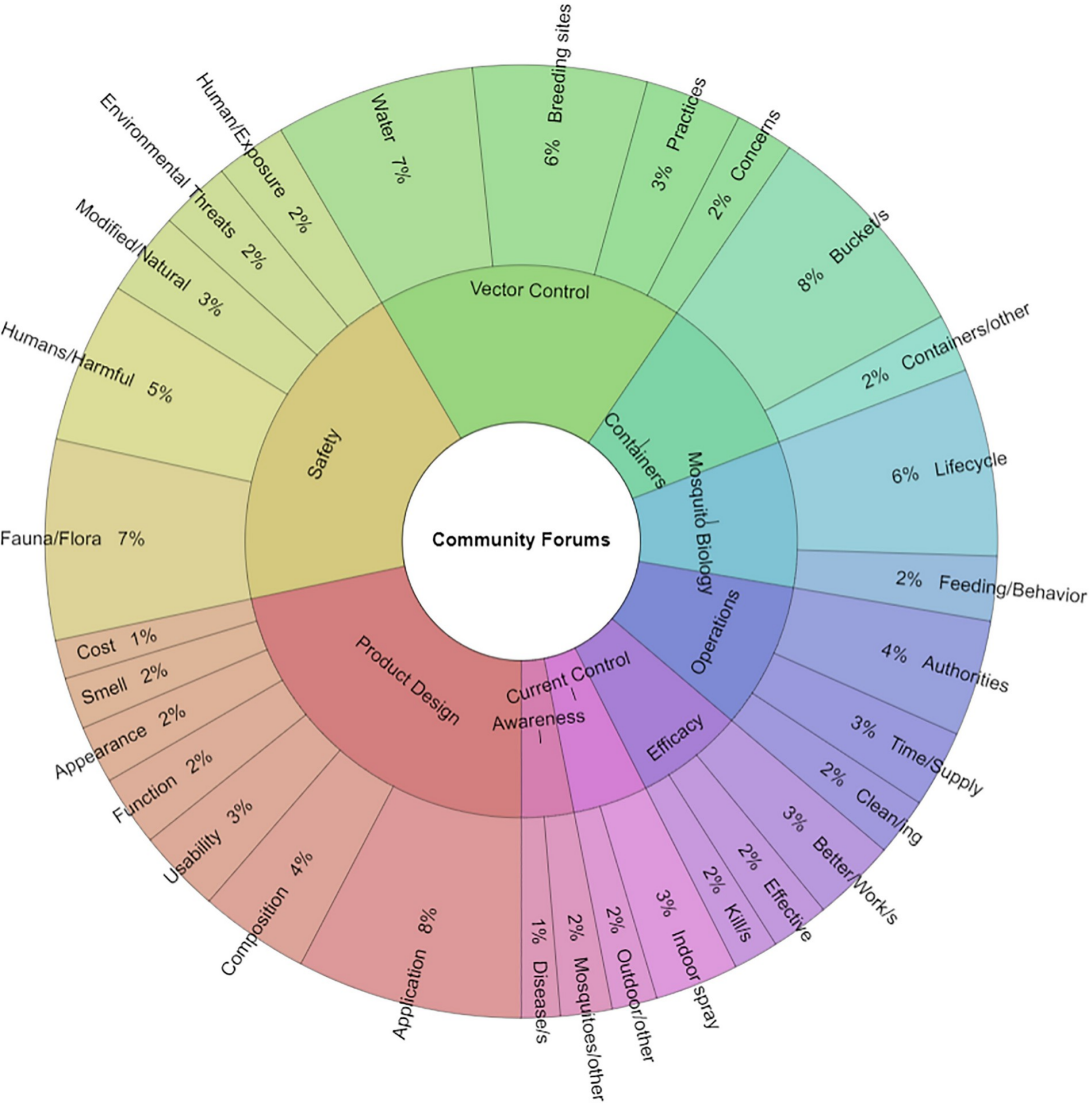
Krona chart detailing words used across the four community engagement forums. Keyword frequency of 457 identified words corresponding to nine themes derived from textual analysis of 5,246 words in the combined transcript from the four community engagement forums. The chart was generated with Krona Excel Template v.2.5 [40].

Transcript analysis on the combined text from community engagement forums, which totalled 5,246 words, was pursued. A summary of repeated words (n = 457) and phrases is illustrated in Fig 5. Frequency and percentage data and word diversity are detailed in S3 Table. Keyword analysis revealed nine themes associated with commonly repeated words: 1) awareness, 2) containers, 3) current control, 4) efficacy, 5) mosquito biology, 6) operations, 7) product design, 8) safety and 9) vector control. The most frequently used words across the four community engagement forums were bucket/s (35 of 457, 7.7%) in the container theme, and application (35 of 457, 7.7%) in the operations theme. In total, 44 of 457 (9.7%) words related to containers, the vessels in which *Aedes* mosquitoes breed, e.g. buckets, barrels, and pots. Of these 44 words, 38 mentions of containers referred to the ovitrap or other bucket that would encourage oviposition. Product design was the largest theme (99 of 457, 21.7% words) and included words such as attracting (11 of 5246 words) and easy (9 of 5246 words). The vector control theme (82 of 5246) included the word water (31 of 82 words) and words referring to the aquatic breeding habitats of mosquitoes (27 of 82 words). The second largest theme was safety (91 of 457, 19.9%), including either environmental (words such as fauna/flora = 31 of 457, 6.8%) or human (25 of 457, 5.5%) safety concerns.

### Household interviews

#### Participant demographics

A summary of interview dates, number of interviews, and number of ovitraps present on the property of each participating household is presented in S4 Table. 29 interviews were conducted in the households of individuals living at 23 of 94 properties (24.5%) on which ovitraps had been placed during the yeast-baited ovitrap field study. Data relating to participant demographics are shown in S3 Fig and reflected, once again, a diverse group of individuals. Females (17 of 29) comprised the majority of participants (58.6%). The most populous age group was those aged 60-years and above (12 of 29, 41.4%). In contrast with the paper survey, engagement forum and census data, Indo-Trinidadians were not the majority race (9 of 29, 31.0%).

*Summary of responses to scripted questions*. A summary of participants’ responses to eight standard questions (S9 File) are summarized following statement of each question below.

***Question 1*. *We would like to know about your experience using the larvicidal ovitraps we are testing*. *How easy or difficult were these products to use*?** 21 of 29 participants (72.4%) agreed that yeast iRNA-baited systems were easy to use. Many of them described the ovitrap set-up as simple, easy to maintain, hassle-free, or straightforward. Notably, they appreciated the fact that it could be placed, treated, and left unattended during a given period while attracting adult females and capturing deposited mosquito eggs. Additionally, 13 of 29 (44.8%) participants commented on its demonstrated efficacy and expressed a willingness to have it permanently placed on their properties.

***Question 2*. *What did you notice*, *if anything*, *about having these larvicidal ovitraps on your property*? *We would like to hear about anything you observed with your senses*, *including what you might have seen*, *smelled*, *touched*, *or heard*.** Most participants (16 of 29, 55.2%) responded to the latter part of this question and stated that they observed nothing in terms of factors affecting their senses. They also made positive remarks about the lack of smell and the product being out of the way. One respondent used this opportunity to express satisfaction in the exclamation “I don’t have to inhale no stupidness!”

***Question 3*. *Ovitraps lure female mosquitos ready to lay eggs*, *and larvicides prevent mosquito larvae from surviving and developing into adult mosquitoes which can bite and carry disease*. *What is your impression of how well the products we are testing functioned as ovitraps (attracting egg-laying females) and larvicides (killing any larvae that developed from the eggs)*?** 17of 29 (58.6%) participants mentioned that the product worked well. In many cases they made statements such as “it’s excellent”, “it doing good”, and “it did its job”. Others noted that they had witnessed egg density during service periods and used this as a basis for their interpretation of product efficacy. Some participants advised a scale-up in operation and commented on the need for additional yeast-baited ovitraps to improve results. Others felt that the system should be complementary to existing vector control tools, for example ultra-low volume (ULV) fogging or spraying for the purpose of eliminating all life cycle stages.

***Question 4*. *If you have used any sort of ovitraps in the past*, *how do the larvicidal ovitraps we are testing compare with others you are familiar with*? *We are interested in hearing about both similarities and differences*.** Most participants (26 of 29, 89.6%) responded that they were not familiar with the ovitrap technique. A minority (4 of 29, 13.8%), who had witnessed its use for general surveillance by MoH/UWI laboratory personnel, declined to comment on the usefulness of its application or practicality. Two participants noted their preference for the size of the bucket (trap) used in these field trials, as it appeared to be larger than the conventional 250 mL ovitrap used by IVCD for surveillance. In both scenarios, individuals associated this with the capacity of the system to accommodate an increased number of eggs due to increased surface area for oviposition. Participants also used this opportunity to mention current vector control practices: indoor and outdoor sprays/aerosols (e.g. local commercial brands), liquid-based chemical pesticides/repellants (e.g. malathion), topical ointments, electronic devices (e.g. vaporizers, electronic bug zapper), and traditional, local or global remedies (e.g. coils, smoke, bleach, engine oils, pitch oil, and vapor rubs).

***Question 5*. *Is there anything about the larvicidal ovitraps we are testing that you particularly liked*? *We are interested in learning about ways our approach to mosquito control might appeal to users more than other types of approaches*.** Participant responses to this question indicated no aversion to the larvicidal ovitraps. 12 of 29 (41.4%) indicated directly that they liked this study approach using the yeast-baited system as a result of its perceived safety towards humans and the environment. Others appreciated its ability to disrupt the mosquito life cycle, thereby reducing population density. One third of respondents (10 of 29, 34.5%) praised researchers’ efforts to educate and inform the community on vector control alternatives and create awareness about mosquito species diversity, ecology, and disease transmission. Some highlighted that the project demonstrated the capability of simple, innovative, “do-it yourself” ideas that could be adopted by householders. They also re-emphasized the benefits of the MoH’s continuous appeals to remove and/or secure water-collection vessels and maintain clean yard spaces/drains.

***Question 6*. *Is there anything about the larvicidal ovitraps we are testing that you did not like*? *We are interested in learning about ways to improve our larvicidal ovitraps*.** 1 of 29 participants (3.4%) indicated a clear dislike for the product. However, the individual noted that it could be deemed acceptable if the aim “to kill all mosquitoes” was met in reference to all species, including nuisance-biting, which was not targeted in this study. Many participants used this question to advise on improved product design or inform researchers on factors to consider in this regard. For instance, one elderly individual thought that the intervention was not convenient for persons who were less able and had some degree of physical disability, while another felt that its use could only be appreciated if the yeast baited-ovitrap system was demonstrated by a researcher. Another participant surmised that the community’s level of satisfaction would have been higher if they had been aware of the ecology of differing mosquitoes prior to ovitrap distribution to account for nuisance species such as *Culex*.

***Question 7*. *If the larvicidal ovitraps we are testing were available for purchase*, *would you buy them*? *If so*, *what do you think a reasonable price for a monthly supply would be*?** 21 of 29 participants (72.4%) mentioned their willingness to purchase the yeast-baited ovitrap system once it became available. Others stated a need for information on mortality rates and were reluctant to decide until the outcome of field trials were known. At this point, participants often listed a set of criteria for purchase, noting that affordability and safety were critical factors. 18 of 29 participants (62.1%) detailed the price they would be willing to pay for a monthly supply. While suggested prices ranged between TT$5-2000/month, the mean price proposed amongst participants was TT$188. When suggesting a price, participants considered their usual expenditures on vector control products, number of ovitraps needed/m^2^, duration of larvicide potency, and the frequency of application. They also inquired about the marketed product and its commercialized package prior to stating an amount.

***Question 8*. *Is there anything else you would like to tell us about the larvicidal ovitraps we are testing*?** 10 of 29 (34.5%) participants re-emphasized their appreciation for the study’s research efforts. 5 of 29 (17.2%) asked questions about the relevance of current vector management techniques, while 4 of 29 (13.8%) highlighted the shortcomings of community practices such as cluttered yard spaces, poor drainage, and propensity to litter/hoard water collection vessels.

#### Transcript analysis

Transcripts from the 29 individuals were analyzed in greater detail. Upon examining the ideas expressed in these transcripts, remarks were categorized into the six codes, (1) information gathering, (2) positive, (3) neutral, (4) negative, (5) knowledge, and (6) recommendations, which were previously described in the community engagement forum analysis. A summary of representative quotes from responses (n = 700) to the eight scripted questions is presented in Table 5. Categorized responses from study participants indicated that one third (231 of 700) were accepting of the technology and made comments that were indicative of their approval. Minimal negative (15 of 700, 2.1%) and neutral (13 of 700, 1.9%) feedback was obtained. In most cases, negativity and indecisiveness related to unfamiliarity with the approach in general and the density of populations of non-*Aedes* nocturnal nuisance biters that were not targeted in this ovitrap study. Most individuals were interested in the use of the ovitraps as a component of integrated vector control strategies. Many sought to relate their expertise by making knowledgeable remarks (157 of 700, 22.4%) while others who became acquainted with the system offered advice (157 of 700, 22.4%) for enhanced utility and public appeal. As expected, some householders (127 of 700, 18.1%) used the interview session as an opportunity to make inquiries and seek clarification to better understand the intervention in greater detail.

**Table 5:** Analysis of the interview transcript data.

Code	Quote	Number of Comments	% of Comments
Positive	• I prefer you all to come all the time because the mosquitoes are less.	231	33.0
	• You’re using this thing here it will lessen down on us having to use spray and all these things so it cutting cost for us in a way too.		

	• It was very effective because I have seen a decrease in mosquitoes by me: a significant decrease.		
Negative	• For us, it’s not a solution because it keeps biting us still.	15	2.1
	• I don’t think it did anything to reduce the number of mosquitoes because it still had the same amount it always has, you know?		
Neutral	• I really can’t say how it would affect me or not.	13	1.9
	• I mean it’s OK.		
Information gathering	• So, all you creating, all you stopping adults?	127	18.1
	• You have to buy the whole system every month? Or it just is a refill you just…?		
	• My concern is the herbicide that you using is it good to be around humans? Is it safe to be around humans?		
Knowledge	• I walk around to make sure it don’t have no life with water and thing, and all kind of thing, but we still have those nasty black mosquitoes you see in the gallery right now. I’m sure they have a lot.	157	22.4
	• …because it is trapping the eggs and it is stopping the adult mosquito so, of course, if there is a decrease in mosquitoes, there will be a decrease in diseases and whatever they spreading, you know?		
Recommendations	• Once it’s affordable.	157	22.4
	• I think if government fund you all—like what you all doing here every month—like if it’s once or twice to go around to the homes and put that, it would be very good health-wise.		
	• You spray once, twice, the spray evaporate so, you don’t really get rid of the mosquito with the spray but this [ovitrap], it come like you building a house to kill the mosquito. You inviting them to kill them.		

The type, number, percentage of total, and representative quotes for each code are shown.

#### Interview themes based on quotes

A total of 121 comments were grouped under the information gathering category. Subsequent analysis of comments in this category highlighted several subcategories of questions (Table 6). Most individuals sought further information concerning the scientific basis behind the approach (20 of 121 questions, 16.5%), followed by price (18 of 121 questions, 14.9%). Comments related to efficacy (12 of 121, 9.9%), research objectives (12 of 121, 9.9%), and application procedure (12 of 121, 9.9%) also appeared frequently. Questions related to commercialization (11 of 121, 9.1%) were also common. Participants also sought information concerning mosquito life history traits (10 of 121, 8.3%), the dosage of yeast used (5 of 121, 4.1%), and vector management (5 of 121, 4.1%).

**Table 6:** Common categories of information gathering questions posed by the interviewees.

Subcategory	Count	Representative Quote
Yeast-baited ovitrap approach	20	The fella only tell me about it but I ask him, I question him “why the brown paper?” and he tell me “well, they does be taking that to see what eggs and how much eggs and whatever.”
Price	18	It would be coming out cheap to the public soon?
Efficacy	12	OK, what you are saying is if I notice that I am having less mosquitoes because of the bucket?
Research/Yeast	12	I just wonder how long the testing will be for?
Application procedure/Operations	12	You could put these things inside your house, your gallery or by your front door? You not putting it in your room but in front by the door?
Commercialization	11	Is the bucket and the paper and whatever?
Mosquito life history traits	10	Well, that is a lot too but how long a mosquito does live for?
Frequency and dose of treatment	5	Every week you have to change it?
Vector management	5	People say keeping your yard clean and all those things does prevent mosquitoes. Is that true?
Conventional methods	4	You mean the spray that I’m spraying?
Availability	3	They don’t sell the little thing?
Safety for humans/animals and environment	3	I have a little puppy in the yard here and if it happen to fall and he lick it, what if it kill him?
Residual activity	2	How long would it be?
Target group	2	So, all you creating, all you stopping adults?
Product design	2	I see you all have a drain hole on it. What is that for? Just in case water overflow?

Typical subcategories of information gathering questions posed by the interviewees. Counts refer to the number of questions in each subcategory (of 121 total questions). Representative quotes from each subcategory are included.

In a similar manner, recommendations were also grouped into subcategories (Table 7). Recommendations related to cost (33 of 60, 55.0%) and product design (15 of 60, 25.0%) were common. Operational recommendations (6 of 60, 10.0%), efficacy (3 of 60, 5.0%) and safety (3 of 60, 5.0%) were also typical.

**Table 7:** Subcategories of recommendations offered by interviewees.

Subcategory	Count	Representative Quote
Cost/Pricing	33	Anything under a hundred dollars would be very good for us.
Product design	15	And if you put a trap with water…. If one portion of… if it have an area that the water in a dark spot and it have an area that water in a bright spot, they would go to the dark spot where the water is.
Operations	6	Well I think you should’ve put more traps.
Efficacy	3	Yes, and if you could get them to kill them one time in the bucket too, eh?
Safety for Humans/ Environment	3	So, we does be kind of scared because he is a baby, you know?

Subcategories of recommendations offered during the interview studies. The number of recommendations (count, out of 60 total) in each subcategory and representative quotations are shown.

#### Keyword analysis

**Fig 6:**
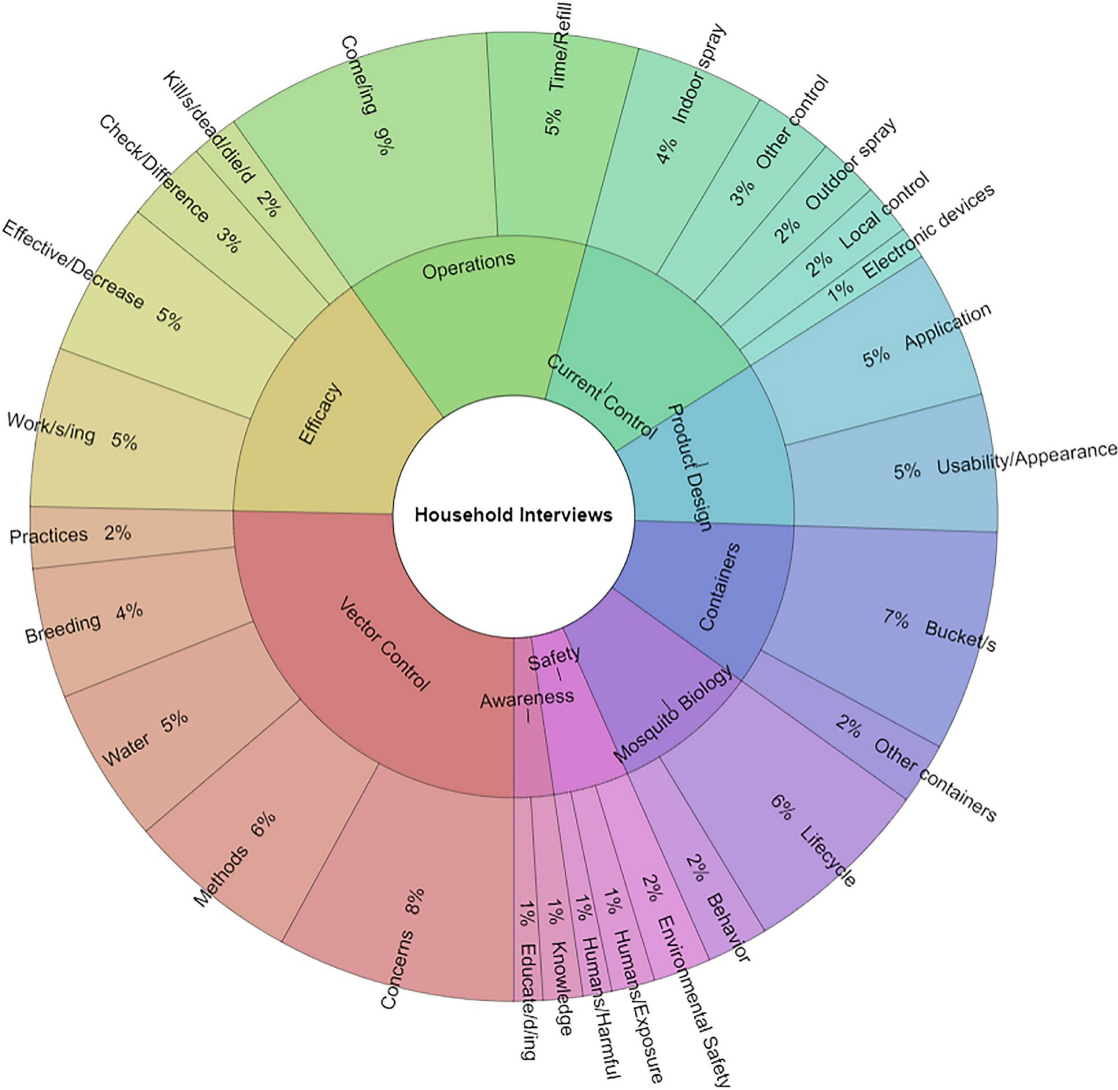
Krona chart summarizing words that were frequently repeated during interviews. Textual analysis revealed words that were frequently repeated by homeowners during the interview study. The chart was generated with Krona Excel Template v.2.5 [40].

Detailed textual analysis of the compiled interview transcripts, which totalled 13,147 words, was performed. Quantifiable usage of keywords and phrases led to the identification of ten themes associated with commonly repeated words (n = 829), which were similar to those identified in the community engagement forums: 1) awareness, 2) containers, 3) current control, 4) efficacy, 5) mosquito biology, 6) operations, 7) product design, 8) safety and 9) vector control. Word frequency and diversity is illustrated in Fig 6 and S5 Table. The most repeated word was come/ing (74 of 829, 8.9%) in the operations theme, followed by concerns (66 of 829, 8.0%) in the vector control theme. In many cases, these words were used in the context of expressing concern that the MoH would visit and service properties more frequently, as well as pleasure that members of the research team were now coming regularly to pursue the ovitrap study and stakeholder engagement investigations. Bucket/s (61 of 829, 7.4%), the main component of the yeast baited-ovitrap system, were also frequently discussed. Interestingly, residents were able to catalogue local control measures (98 of 829, 11.9%), for example aerosolized pyrethroids and cultural practices such as the use of engine oil. The vector control theme had the highest number of keywords (210 of 457, 25.1%), while the safety theme was only mentioned 36 of 829 times (4.3%) across the 29 household interviews.

## Discussion

This study assessed the willingness of residents across communities in Trinidad to integrate yeast iRNA-baited ovitraps into treatment regimens around their homes. It also explored the impact of mosquito abundance on participants’ life by cataloging their familiarity with vector mosquito management strategies through an assessment of their level of working knowledge, engagement in preventive practices, and acceptance of alternative control interventions. Results supported observations from a previous study [34]—which highlighted community-stakeholders’ willingness to adopt yeast iRNA larvicides—as participants valued efforts to control mosquitoes and mitigate the spread of associated diseases. The present study extended the initial investigation [34] by exploring stakeholders’ interest in using the larvicides in ovitraps which had been placed on their properties. Stakeholders provided useful advice regarding ovitrap design, distribution, and management. The study informed stakeholders of the new intervention, simultaneously allowing them to play a direct role in the optimization of the novel technology, which was broadly accepted as a promising new control strategy.

### Paper surveys reveal insight into stakeholders’ baseline knowledge, current practices and willingness to adopt alternative vector control approaches

Paper surveys proved to be an important canvassing tool for assessing study participants’ baseline knowledge of mosquitoes, awareness of the pathogens that mosquitoes transmit, and existing means of vector control. Responses indicated that participants had a working understanding of mosquito-borne illnesses, patterns of disease transmission, and efforts to reduce mosquito populations to mitigate the spread of arboviral illnesses. As previously observed [34], a majority of respondents once again agreed that treating water (Fig 3B), and using larvicides (Fig 4B), would reduce local mosquito populations, while now also indicating that ovitraps (Fig 4F) would contribute to mosquito reduction efforts. Individuals were still more likely to use a specific control method if they believed it would reduce disease transmission or the number of mosquitoes [34]. However, responses showed that participants were more likely to remove standing water around their homes (83.4%; Fig 3C) than to use larvicides (32.4%; Fig 4B) or ovitraps (8.8%; Fig 4F). This may be due to their baseline knowledge and/or familiarity with each control method at the onset of this investigation, especially since larvicides are the most commonly applied control approach through MoH-IVCD operational activities, while the use of ovitraps is limited, at present, to general surveillance. Targeted educational campaigns by the MoH routinely encourage individuals to remove any potential *Aedes* breeding sites. As this method of source reduction is relatively easy to conduct and bears no cost to the householder, it is not unexpected that respondents would be more aware of its utility. It is interesting to note, however, that despite 93.4% of respondents agreeing that treating water would result in a reduction of disease prevalence (Fig 3B), this sentiment did not translate into a similar pattern of current larvicide use (32.4%; Fig 4B). Possible reasons that may account for this observation, could be: (1) concerns for the potential adverse impacts of existing chemical insecticides on human health and the environment, as conveyed by participants in community engagement forums (Fig 5 and Tables 3 and 4 and S3) and household interviews (Fig 6 and Tables 5–7 and S5), (2) lack of confidence in efficacy, which may be due to resistance to existing pesticides documented in previous Trinidad studies [19, 20] and householder observations following repeated use of aerosolized sprays (Table 5), and (3) cost.

Irrespective of the differences among respondents’ beliefs and current practices, responses indicated a general willingness to use existing control methods (Fig 4C) and/or adopt new technologies (Fig 4H), which further translated into purchasing motivation (Fig 4D and 4G). Although rationale behind participant responses was not directly revealed through paper survey assessments, correlations between responses to specific questions could be identified and tested for statistical significance. For example, those willing to buy a larvicide had also reported larvicide use in their household during the last year. Familiarity with the control methodology was probably, therefore, a factor in purchasing decisions. Paper survey participants suggested mean monthly prices of TT$65.90/larvicide and TT$69.27/ovitrap, indicating that they perhaps did not recognize a substantial difference between the two technologies. As noted above, this finding may also reflect a general lack of familiarity with larvicides and lethal ovitrap technology, which could be better emphasized in future educational campaigns. Education by itself, however, is insufficient to generate behavioral change, which requires at the minimum sustained face-to-face interaction over prolonged periods to demonstrate how a community’s actions on day one can impact upon an outcome at a later date [41].

### Community engagement forums increase public awareness and engagement

Community perspectives are a valuable but often missed dataset. The team considered the pivotal role that this stakeholder group plays in bridging the gap between science and its application in solving real-world problems by conducting engagement forums as a platform to facilitate exchange of information between both parties. Additionally, these events also introduced participants to yeast iRNA technology—a novel class of mosquito larvicides—and its utility in manipulating *Aedes* aquatic breeding sites through a lure-and-kill approach [25]. This format generated a good deal of discussion, as evidenced by the large number of information gathering questions raised during the forums (Table 3). The interactive nature of the forums prompted respondents to be direct about their perceptions and concerns. Notably, it became relevant for researchers to highlight the key morphological and behavioral characteristics of nocturnal nuisance-biting *Culex* species vs. *Aedes* mosquitoes, the focus of this study. Further discussion centered on participants’ recommendations on product design (Fig 5 and Tables 4 and S3).

Participants’ interest in mosquito biology and life history traits indicated knowledge gaps in their general understanding of mosquito ecology. Regardless, participants appeared to be willing to invest greater efforts into mosquito control around their property and to take a more proactive approach. Traditionally, vector management in Trinidad is based on the operations of perifocal officers from the MoH-IVCD [42]. Community engagement forums, however, highlighted that stakeholders appreciated the value of a combined effort involving both residents and the MoH-IVCD in integrated mosquito control programs. They also discussed limitations of currently approved larvicides [21] and the critical need to develop alternative interventions that might become useful new additions to integrated mosquito control programs. Many participants understood the concept of insecticide resistance as detailed by their numerous comments on the efficacy of sprays and chemical fogs (Fig 5 and S3 Table). A large proportion of comments were associated with product application and operational issues, demonstrating overall enthusiasm for optimizing this technology for use around their homes. Engagement participants were willing to pay double (TT$68.18/month) for a yeast iRNA-baited ovitrap over larvicide use in other containers on their properties [34], a notable finding, particularly given that the MoH-IVCD has traditionally been responsible for mosquito surveillance and control in Trinidad and Tobago. This willingness to pay out of pocket may correlate with the perception, noted amongst some forum participants, that IVCD visits to residences should occur more frequently (S6 File). Nevertheless, a number of participants also noted that they believed that the government should be involved, and they made operational suggestions for effective use of the ovitraps (Table 4).

### The value of household interviews during implementation of novel vector control strategies

Empowering communities is a critical component of developing sustainable vector control programs [41]. Given that the household interviews conducted in this investigation occurred in parallel to a yeast-baited ovitrap field trial, participating interviewees had actually witnessed the use of ovitraps on their properties. Interview sessions provided researchers with the opportunity to discuss ovitrap use with the participants, while also facilitating general dialogue related to mosquitoes and their control. These interactions, which occurred initially at the commencement of the ovitrap field trial, followed by weekly encounters during ovitrap maintenance periods, served to develop and strengthen public engagement between researchers and the community.

Most participants acknowledged the ease with which the yeast-baited ovitrap could be installed and found it straightforward to integrate ovitrap use into their existing control regimens. Residents’ observations during the field trial enhanced their ability to discuss vector management strategies. Frequent topics of discussion during the interviews included ovitrap efficacy and safety (Fig 6 and Tables 6 and 7 and S5) together with the merits and limitations of existing control practices. In terms of the safety theme, the practical experience of the larvicidal baited ovitrap satisfied safety concerns, which were reduced in the keyword analyses of the interviews (4.3% of total words) as compared to the community engagement events (19.9% of the keywords). Observation of the yeast-baited ovitrap technology also allowed participants to offer detailed advice and recommendations on product design (Table 7). Moreover, sustained face-to-face interactions with the researchers provided opportunities for residents to understand and accept the scientific technology implemented in the yeast-baited ovitrap, with the majority expressing a general willingness to adopt it as an alternative method of mosquito control on their properties (Fig 6 and S5 Table). Participants suggested that they would be willing to pay a mean of TT$188/month for a larvicidal ovitrap—an amount three times higher than either of those suggested by the paper survey and engagement forum participants—illustrating how residents benefited from an enhanced operational understanding provided through ongoing engagement. Nevertheless, some participants still preferred that the government subsidize the intervention, with one participant even suggesting that the government pay the researchers to come to their properties (Table 5). This suggestion appeared to represent a more general sentiment amongst some study participants that the government should pay for the costs, but that it would be good if government investment in mosquito control products would coincide with more frequent visits to their properties. Regardless of whether such devices were to be purchased for use by the MoH-IVCD, by private citizens, or both, it should be noted that at this stage of the research project, the ovitraps under evaluation are simply at the prototype stage and not available for commercial use, a point that was made clear to the participants when relevant discussions arose during the engagement forums or interviews. Questions of cost were only included to prompt further discussion and consideration of this topic, which did occur.

Given the prevalence of mosquito-borne diseases in Trinidad [43], it was anticipated that participants in the simulated field trials would have a reasonably good working knowledge of mosquitoes, mosquito ecology, and vector-borne pathogens. This expectation was consistently observed throughout the household interview study, as participants made frequent reference to morphological and behavioral characteristics typical of *Aedes* mosquitoes (Tables 5 and S5). Household interviews illustrated that residents were aware of mosquito-borne illnesses as a public health issue. Importantly, interactions between stakeholders and vector control staff during the interview study period may have helped to improve MoH-IVCD relations in study site areas. These interactions highlighted the importance of stakeholder involvement in vector control. It became apparent to many household participants that government-based efforts were not an end-point to vector-related issues. Study participants realized that common societal practices contribute to environmental conditions that encourage *Aedes* breeding, which, ironically, then require increased perifocal control operations by the MoH-IVCD [42].

This project provided an avenue through which participants perceived that they had gained a voice in local vector control efforts, an important component of long-term partnerships that legitimizes and adds value to programs [41]. Stakeholders were eager to utilize chemical-free alternatives that are determined to be safe, effective, and available. Moreover, responses to questions regarding mosquito traits and life history demonstrated to residents the benefits of targeting the correct vector species. The exchange of information on mosquito ecology equipped participants with a basic understanding of mosquito species diversity in their area, allowing them to make more informed recommendations regarding product development and optimization.

### Comparison of assessment tools

To better ensure the acceptance and success of intervention methods, engaging in open, transparent, and collaborative communication with communities is a guiding principle in the implementation of new vector control initiatives [41, 44–46]. The success of intervention methods can be dependent on public approval and cooperation, a notable example being the 79% reduction in *Ae*. *aegypti* juvenile stages together with zero reported dengue cases observed in a two-year Cuban study [47]. A current issue is that scientific researchers are not unified in what is deemed an acceptable amount of information and knowledge to provide to community members [46, 48]. There is a need for benchmarks from public health organizations outlining what constitutes acceptable support from, and engagement with, the community [46]. Additionally, information is needed to understand what study methods are best suited to inform and engage community members to gain their input and address any opposition to the project.

At the commencement of this project, paper surveys were distributed prior to any education on mosquito control methods or the specific product. As noted, the survey was able to function as a tool to obtain an initial assessment of existing stakeholder knowledge of mosquito-borne disease and practices of members in the community. Utilizing this study tool allowed for many individuals to be sampled in a quick and efficient manner. However, surveys are generally designed as a tool to gain knowledge of people’s beliefs and behaviors and do not offer insight into individual’s motivations or reasons for beliefs, practices, and behaviors. As such, it was essential that community engagement forums were also conducted prior to the commencement of field trials. As an assessment tool, engagement forums are better suited for capturing the motivations and factors behind individual attitudes. Following these engagement events with household interviews allowed for a more comprehensive assessment of community acceptance of the product before and after interacting with it. A primary advantage of the public engagement forums was that these events allowed the attitudes of many people to be garnered at one timepoint, allowing for general insight into the desires and concerns of the community as a whole. Additionally, the group format of the engagement forum permitted individuals to ask questions as they arose, while also considering the sentiments and questions of others. Importantly, the community engagement forums appeared to empower participants by providing a platform in which they could participate and contribute meaningfully to the discussion, development, optimization, pricing, and utility of the product appropriate to their local conditions [41]. This is essential to the development of a positive relationship between researchers and the public. A limitation of the forum-based approach is that it only assessed opinion and perception of the technology based on information that participants received during events, rather than personal experience with the yeast iRNA-baited ovitrap. This may have contributed to participants’ initial lack of understanding as evidenced through the large number of information gathering questions (Table 3). Additionally, the engagement forum settings may have served to amplify the opinions of those who were more vocal. Those who preferred to maintain anonymity may have been hesitant to speak up in fear of identification or conflict with others’ opinions.

Face-to-face interviews were able to compensate for some of the limitations of the community engagement events as well as to provide further useful insight. Since interviews were conducted after participants had engaged with the yeast iRNA-baited ovitrap, individuals were able to provide more thorough and informed product-specific feedback than in the community engagement events. Additionally, while each individual may not have had the chance to answer each question in the engagement events, the interview afforded the opportunity for each interviewee to answer every question. Further, interviewers were able to better capture non-verbal cues (nods, shrugs, eye rolls etc.) and probe respondents to elaborate. Interviews also created a more comfortable setting for those who may have felt shy in a group setting. These individuals may have felt freer to honestly convey their opinions. They were also less likely to be biased by the sentiments of other individuals than during the community engagement events. Interviews tended to yield less recommendations and questions about the product, with subjects demonstrating greater knowledge of the intervention (Table 5), most likely due to their direct exposure to use of the product. One of the limitations with the interview method as an assessment tool, however, is that it resulted in a smaller sample size since it captures the opinions of fewer individuals thus, becoming less representative of the population as a whole. In addition, it generated a demographic bias in which the participants who agreed to respond were predominantly older in age (S3 Fig) since they were more likely to be present at home during the daytime when researchers conducted ovitrap servicing.

The use of all three assessment tools provided greater insight into the knowledge, practices, and perceptions of community stakeholders toward yeast iRNA-ovitraps. Paper surveys, although anonymous, permitted large-scale sampling and quantitative analysis while engagement forums and interviews facilitated dialogue between participants and researchers. Transcript analysis of these exchanges identified word frequencies and comments (Figs 5 and 6), which could be interpreted further. Community forums and household interviews also strengthened public engagement in a manner that allowed for participants to learn about the product, as well as ask questions and receive immediate information. Concerns and recommendations raised at engagement forums were subsequently addressed in face-to-face household interviews. Utilizing all three assessment tools allowed for the identification of key concerns among community stakeholders by noting intersectionality in questions and sentiments expressed in responses. It also permitted analysis of general changes in attitude prior to, and following, use of the product.

### Community perceptions of vector control technologies

The analysis of community perceptions of arbovirus vector control methods and technologies is generally regarded as a critical aspect of successful integrated mosquito control programs [49]. The management of vector-borne diseases is nevertheless associated with ethical challenges, particularly with respect to the introduction of new control innovations. To address this, the WHO recently developed guidance on ethical issues associated with vector control implementation [49], noting that control of vector-borne diseases, which disproportionately impact the poorest populations of the world, is influenced by social determinants of health, for example age, gender, and socioeconomic status, and must be carefully administered. Moreover, vector management is typically dependent on collective actions by communities, and may or may not involve the knowledge and consent of the individual [49]. For these reasons, the WHO prioritizes community engagement, which it defines as “a process of developing relationships that enable stakeholders to work together to address health-related issues and promote well-being to achieve positive health impact and outcomes [50].” Involving community members to ensure that their issues and concerns are heard and considered, and that the research team values the establishment of a partnership with the community, which has been empowered to impact the course of the studies, are key components of effective engagement [49]. As detailed herein, these activities have been prioritized in Trinidad, where study participants have gained critical knowledge of the new intervention and ultimately demonstrated general support and enthusiasm for yeast interfering RNA ovitrap technology. Importantly, study participants shared many suggestions for improvement of yeast RNAi-based ovitraps, for example the development of long-lasting yeast larvicides, as well as suggestions that the team develop methods to control additional species of mosquitoes and adults, suggestions that the research team has incorporated into ongoing analyses of integrated RNAi mosquito control tools.

The WHO [49] acknowledges the difficulty of evaluating community acceptance of innovative new population-based control methods that may involve risks of unknown implications for the environment or health, or which may have the potential to induce irreversible changes to mosquito populations that are likely to spread across national borders [49]. The guidelines note, for example, that Wolbachia-based control strategies involve the artificial infection and release of mosquitoes with maternally inherited bacteria for the purposes of population suppression or reduction of pathogen transmission and review the critical community engagement efforts that have accompanied evaluation of this technology [49]. Likewise, transgenic-based control strategies, such as gene drives, typically focus on the dissemination of genetic traits that reduce vector reproduction or pathogen transmission [44–49], and effective community engagement has accompanied the scientific and ethical evaluation of this emerging technology [49]. Likewise, the potential implementation of RNAi-based mosquito control intervention necessitates effective community engagement, as detailed here and in a related study [34]. It should be noted, however, that although yeast-based RNAi larvicides involve genetic manipulation of yeast, the yeast is heat-killed prior to use, and so use of this intervention does not involve the release of live genetic model organisms or heritable genetic modification of natural populations of mosquitoes, an important distinction with respect to gene drive strategies. Moreover, although the use of yeast involves reduction in mosquito numbers, the yeast does not induce the spread of a genetic trait throughout the mosquito population [32]. Such distinctions may impact societal acceptance of the technologies involving GMOs, a topic which has not yet emerged as a primary concern with respect to RNAi-based yeast larvicides in Trinidad, as noted here and in another recent study [34], but one which has been debated with respect to emerging transgenic mosquito control technologies [44–49]. Further engagement activities within Trinidad and Tobago and beyond can continue to investigate stakeholder acceptance of yeast RNAi technology at other locations within Trinidad and Tobago and more globally.

## Conclusions

Public awareness is the first step to mitigating the spread of vector-borne illnesses. This study identified the attitudes of community stakeholders in Trinidad toward current and alternative mosquito control practices. The majority of respondents indicated support for alternative technologies—specifically larval lethal ovitraps and yeast-iRNA larvicides. This study highlighted the importance of initiating community relationships at the commencement of a project, in addition to emphasizing the need for continuing public engagement activities as the program progresses. It also demonstrated the value of combining different assessment tools to obtain a comprehensive community-wide picture. The long-term objective of this research is to incorporate stakeholder-accepted RNAi-based larval lethal ovitraps into integrated mosquito control programs. Achieving this goal involves the development of scalable commercial larvicide formulations and demonstration of larvicidal activity in ovitraps evaluated in large-scale field trials to be conducted in Trinidad and other parts of the world. Longitudinal studies, particularly those which involve interviews with individuals residing in homes at the study sites, will enable continued public engagement with stakeholders and help ensure the success of this novel mosquito control intervention.

## Supporting information

S1 File Study information sheet—mosquito larvicidal ovitrap survey. This information sheet, which provided background information on the research study, was distributed to participants prior to their completion of the paper surveys.
(PDF)

S2 File Mosquito larvicidal ovitrap survey. The printed paper survey, which included an optional demographics section, that was distributed to participants during this study.
(PDF)

S3 File Study information sheet—larvicidal ovitrap community engagement forum. This information sheet was distributed to participants just prior to the community engagement forums.
(PDF)

S4 File Demographic questions for community engagement forum participants. Engagement forum participants were given an opportunity to self-report demographic information. Completion of the demographic information form was optional.
(PDF)

S5 File Script—introduction for community engagement forum. This script was used by the moderators to introduce the community engagement forum.
(PDF)

S6 File Script–larvicidal ovitrap community engagement forum study. This series of questions was posed by the moderators during the engagement forums.
(PDF)

S7 File Study information sheet—larvicidal ovitrap trial participant feedback interview study. This information sheet was provided to interview participants prior to conducting the interviews.
(PDF)

S8 File Demographic questions to accompany interview—household participant feedback study. This optional demographic sheet was provided to interviewees.
(PDF)

S9 File Script–larvicide trial participant feedback study. This series of questions was posed by the interviewer during the interview study.
(PDF)

S1 Fig Concept map of study. A summary of the three approaches used for data collection is provided.
(PDF)

S2 Fig Demographic information for participants at community engagement forum events. Data for participants’ age, gender, race and education are shown.
(PDF)

S3 Fig Demographic information for participants in household interviews. Gender, age, race and education of householder interviewees are shown.
(PDF)

S1 Table Paper survey collection in Trinidad. The counts and percentages of the total surveys collected are detailed for major areas across Trinidad.
(PDF)

S2 Table Summary of engagement forum events and attendance. The location, date and number of attendees for each community engagement forum are detailed.
(PDF)

S3 Table Summary of word count analyses of the community engagement forum responses. Word count analyses revealed ten commonly repeated words. Commonly repeated words, the number of times that the words appeared (among a total of 5,246 words across the four community engagement forums), and quotes that exemplify the context in which the words were often used are shown.
(PDF)

S4 Table Summary of in-field ovitrap household interviews. The household number, number of individuals interviewed at each household, interview dates, and number of ovitraps placed at each residence are detailed.
(PDF)

S5 Table Summary of themes from word/phrase count analysis of household interviews. Word count analyses revealed ten themes associated with commonly repeated words. Common words associated with each theme, the number of times that the words appeared (among a total of 13,147 words across 29 individual interviews), and quotes that exemplify each theme are shown.
(PDF)
