# Supplementary material for: Assessment of Trinidad community stakeholder perspectives on the use of yeast interfering RNA-baited ovitraps for biorational control of Aedes mosquitoes
Source: PLoS One. 2021 Jun 29;16(6):e0252997. doi: 10.1371/journal.pone.0252997 (PMC8241094; doi:10.1371/journal.pone.0252997)
Supplement: S2 File — The printed paper survey, which included an optional demographics section, that was distributed to participants during this study. (PDF) [file pone.0252997.s002.pdf]

Mosquito Larvicidal Ovitrap Survey

Please mark the circle which most closely represents your agreement or disagreement with the following statements about mosquito larvicides, pesticides that kill mosquito larvae, and ovitraps, black cups that attract females ready to lay eggs.

	Strongly disagree	Somewhat disagree	Neither Agree nor Disagree	Somewhat agree	Strongly agree
Diseases such as dengue, Zika, chikungunya, and yellow fever are caused by viruses transmitted by adult mosquitoes.	☐	☐	☐	☐	☐
Treating water where mosquitoes breed will reduce disease transmission.	☐	☐	☐	☐	☐
I, or someone in my household, have tried to reduce the number of mosquitoes by removing standing water around my home.	☐	☐	☐	☐	☐
Use of larvicides will help reduce the number of mosquitoes.	☐	☐	☐	☐	☐
I, or someone in my household, have used larvicides around my home in the past year.	☐	☐	☐	☐	☐
I would be willing to use larvicides to treat water around my home in the next year.	☐	☐	☐	☐	☐
I would be willing to buy a larvicide.<br>• *If you strongly or somewhat agree:*<br>An affordable amount for our household to spend on larvicides each month would be: $__________	☐	☐	☐	☐	☐
Use of ovitraps will help reduce the number of mosquitoes.	☐	☐	☐	☐	☐
I, or someone in my household, have used ovitraps around my home in the past year.	☐	☐	☐	☐	☐
I would be willing to use larvicides to treat water around my home in the next year.	☐	☐	☐	☐	☐
I would be willing to buy an ovitrap.<br>• *If you strongly or somewhat agree:*<br>An affordable amount for our household to spend on ovitraps each month would be $______	☐	☐	☐	☐	☐
If genetically modified organisms (GMOs) were known to be safe and effective larvicides, I would support their use in ovitraps.	☐	☐	☐	☐	☐

Optional: Please tell us more about your household and about you:

In the past two years, how many times has someone in your household had dengue, Zika, chikungunya, or Yellow fever? If you cannot remember exactly, it is alright to estimate: _____

How many adults 18-59 years of age live in your household? ______

How many children under the age of 18 years live in your household? _____

How many adults 60 years of age or older live in your household? ______

What is your gender? *Male* *Female*

What is your age in years? *<20* *20-29* *30-39* *40-49* *50-59* *>60*

What is your race? *Afro-Trinidadian* *Indo-Trinidadian* *European Descent* *Chinese descent* *Mixed descent* *Other*

Indicate the highest level of formal education you completed or in which you are presently enrolled: *Tertiary* *Secondary* *Primary* *None*
