# Supplementary material for: Assessment of Trinidad community stakeholder perspectives on the use of yeast interfering RNA-baited ovitraps for biorational control of Aedes mosquitoes
Source: PLoS One. 2021 Jun 29;16(6):e0252997. doi: 10.1371/journal.pone.0252997 (PMC8241094; doi:10.1371/journal.pone.0252997)
Supplement: S3 File — This information sheet was distributed to participants just prior to the community engagement forums. (PDF) [file pone.0252997.s003.pdf]

*Study Information Sheet *

Larvicidal Ovitrap Community Engagement Forum

You are invited to participate in a community engagement event about the use of mosquito larvicides and ovitraps in Trinidad. The people of Trinidad can contract serious diseases through the bites of mosquitoes, and a research project being conducted here will investigate new ways of preventing mosquitoes from transmitting infections.

This study is being conducted by Dr. Azad Mohammed (The University of the West Indies at St. Augustine, Trinidad and Tobago), as part of a larger research project led by Dr. Molly Duman Scheel of Indiana University School of Medicine-South Bend, in collaboration with Dr. David W. Severson and Nicole Achee of the University of Notre Dame (United States). You are invited to participate in this study because you are an adult resident of Trinidad. If you have any questions about this study, please contact Dr. Molly Duman Scheel at (574) 631-7194 (country code 1) or mscheel@nd.edu. For questions about your rights as a research participant, to discuss problems, complaints, or concerns about a research study, or to obtain information or offer input, contact the IU Human Subjects Office at 317-278-3458.

This study consists of a presentation of information about our larvicidal ovitrap study, followed by an open forum discussion, during which you can ask questions about the study. We will also ask questions intended to solicit your input about the larvicidal ovitraps we are testing. As our research project pursues new ways of preventing mosquitoes from carrying disease, it is very important for us to consult adults in households where larvicidal ovitraps may be used. We hope to learn about your thoughts, feelings, and opinions regarding the use of our larvicidal ovitraps.

Participation in this study is voluntary and you may choose to stop participating in this study at any time. This study consists of an information session, during which you will learn about larvicidal ovitraps. This will be followed by a question and answer session. You will have the opportunity to ask questions, and to express your thoughts about the study, positive or negative, in answer to our questions. We anticipate that this community engagement event will take 1-2 hours.

If you agree to participate in this study, the investigating researcher will ask the group questions that will promote discussion. The investigating researcher will also provide you with the opportunity to ask questions and express your thoughts, feelings, and opinions. Your spoken responses will be audio recorded. You will also be asked to enter some basic information about yourself and your household on a paper form, which will be collected by the interviewing researcher.

Participating in this study may not provide any direct benefit to you. The knowledge gained from this study will be used as part of a large research project that may result in the development of new mosquito control devices, larvicidal ovitraps, ultimately intended to disrupt the transmission of human disease. We do not envision any significant risks related to participation in this study. Privacy with respect to information you will share with us if you participate in this study will be protected by the investigators. Audio recordings will be converted to digital transcripts which will not contain any individually identifying information, and the original recordings of your voice will be deleted. Your name and other information which would allow you to be identified as an individual are not being collected in this study, so your spoken and written responses will not be attributable to you.

Thank you for agreeing to participate in our research. Before you begin, please note that this research is for residents of Trinidad over the age of 18; if you are not a resident of Trinidad, and/or you are under the age of 18, please do not participate in this study.
