# Supplementary material for: Assessment of Trinidad community stakeholder perspectives on the use of yeast interfering RNA-baited ovitraps for biorational control of Aedes mosquitoes
Source: PLoS One. 2021 Jun 29;16(6):e0252997. doi: 10.1371/journal.pone.0252997 (PMC8241094; doi:10.1371/journal.pone.0252997)
Supplement: S5 File — This script was used by the moderators to introduce the community engagement forum. (PDF) [file pone.0252997.s005.pdf]

Introductory Script for Community Engagement Forum

The following script was presented by a moderator for each Community Engagement Event.

Introduction

Do you know how many mosquito species exist in Trinidad and Tobago? [Pause for feedback]

Ok, there are 178 species. Many exist in the forest and humans never encounter them. However, some of them you will be familiar with such as the famous *Aedes aegypti* which will be the topic of our discussion today.

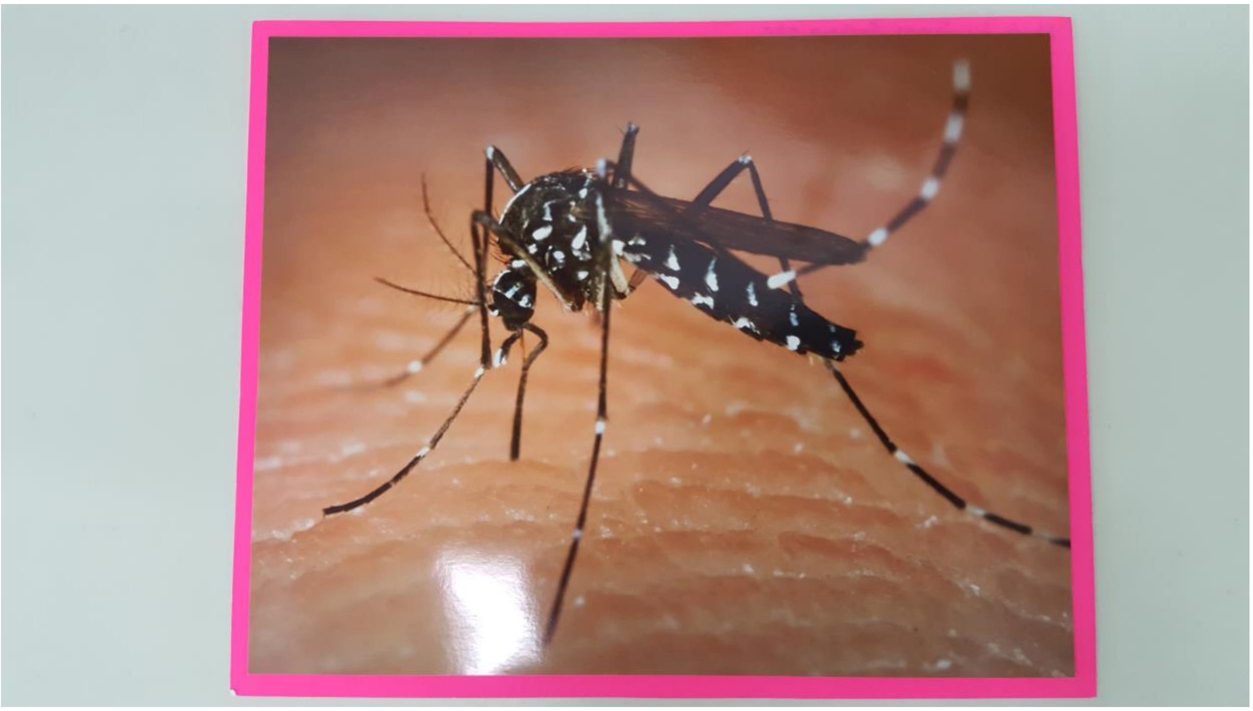

Fig 1. Demo picture of mosquito. Photograph of an *Aedes albopictus* mosquito mounted on colored bristol board. (Photo credit: James Gathany of the Centers for Disease Control)

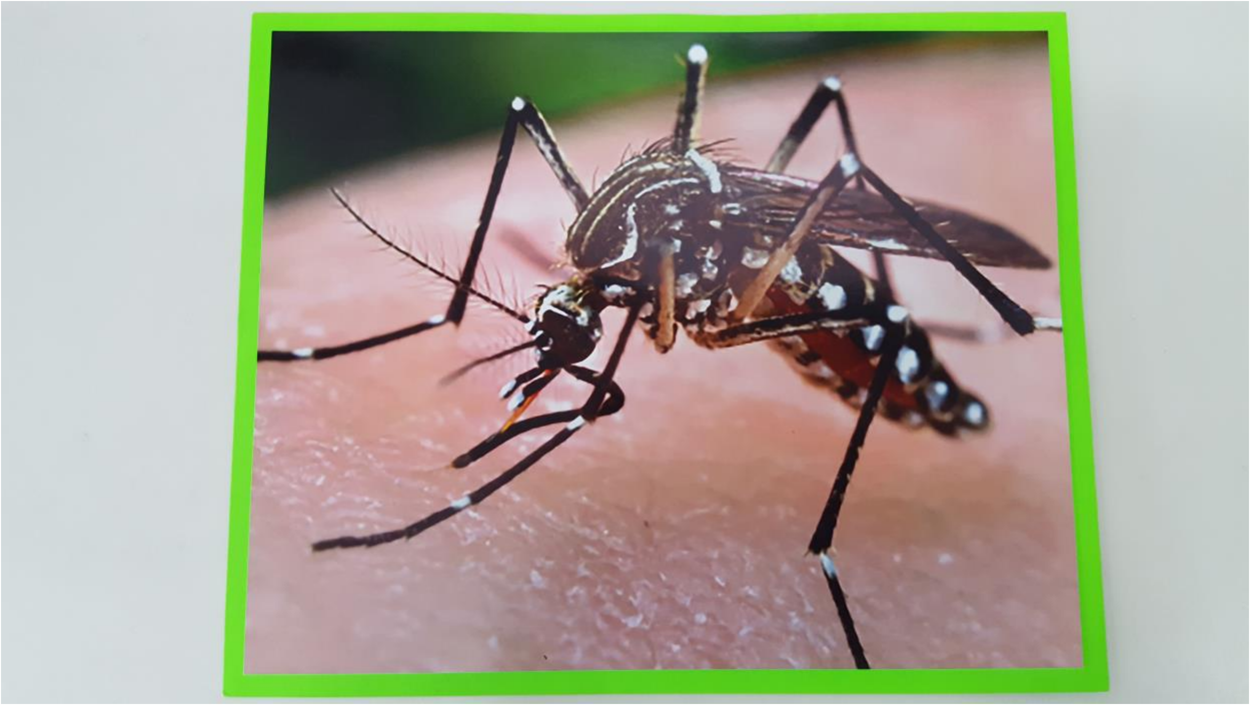

Fig 2. Demo picture of mosquito. Photograph of an *Aedes aegypti* mosquito mounted on colored bristol board. (Photo credit: James Gathany of the Centers for Disease Control)

Aedes aegypti is a mosquito that spreads diseases, including dengue fever, chikungunya, Zika and yellow fever in Trinidad and Tobago. Are you familiar with any of these diseases?

Those who have suffered or know someone that has contracted these illnesses caused by viruses transmitted by mosquitoes, know of the symptoms, which include high fever, rash, and muscle and joint pain, headache, fatigue, and vomiting and complications from these diseases can even result in death.

With no commercially available medications or vaccines for most of these diseases, targeting the mosquito is the primary means of disease prevention.

Most of the existing methods for mosquito control rely on the use of chemical pesticides. For example, larviciding, the addition of chemical insecticides to water in which mosquitoes breed, is critical for controlling Aedes mosquito larvae.

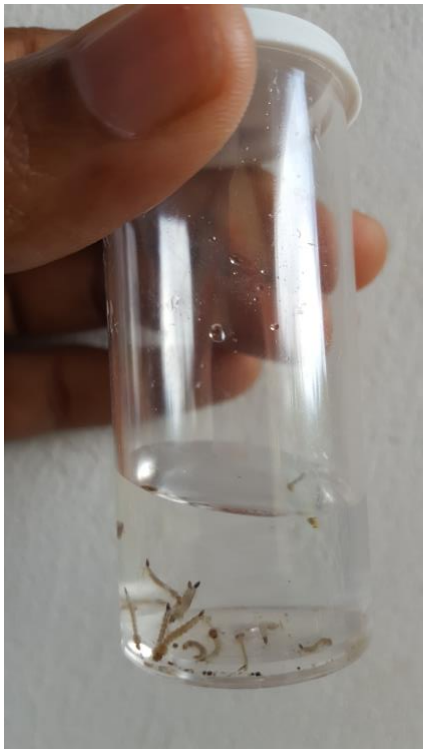

Fig 3. Demo picture of larvae Photograph of field collected *Aedes* larvae

There are many different larvicides currently available on the market, but most are chemical based.

Sadly, resistance to these insecticides has begun to emerge, and there are rising concerns about harmful effects of these pesticides for humans and non-target species like bees and other pollinators which are important for the agricultural sector and environmental biodiversity.

We are developing a new pesticide strategy based on biological agents. It involves genetically modifying baker's yeast that expresses larvicides that interrupt mosquito development.

The technique used in these larvicides is designed to match mosquito genes, not the genes of humans or other non-target organisms. This means that this novel larvicide is designed to kill only mosquitoes and nothing else. When the mosquito larvae eat the yeast, they die.

The yeast makes the larvicide when it is grown. This allows for simple and cost-effective larvicide production.

We are studying ways to deliver the yeast to mosquito larvae. One method is to put the yeast in ovitraps. Ovitraps are a special type of dark container that are lined with paper and used to attract mosquitoes that are ready to lay eggs.

Demonstration of eggs and ovitrap

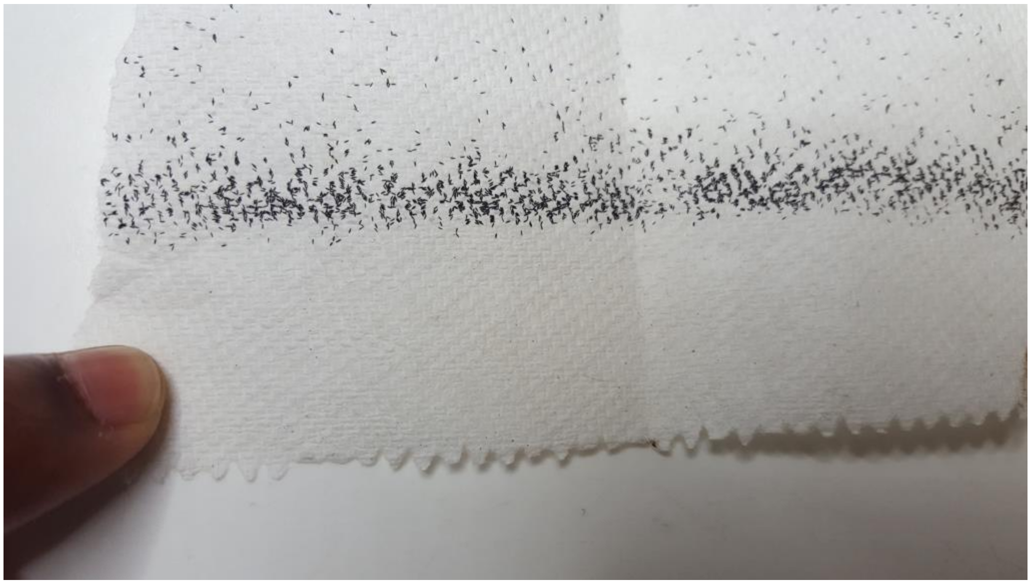

Fig 4. Demo of paper with mosquito eggs. Example of *Aedes* mosquito eggs on paper towel displayed at engagement

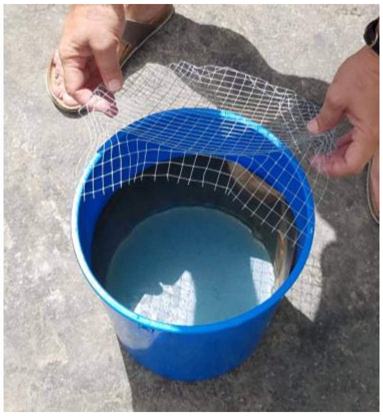

Fig 5. Demo of ovitrap. Example of ovitrap displayed at engagement

Chemical pesticides are currently included in ovitraps to kill mosquitoes. Instead of using these chemicals, we plan to add our yeast larvicides to the traps.

Our initial studies show that the yeast actually attracts female mosquitoes to lay their eggs in the traps. When the larvae hatch from the eggs, they eat the yeast and die. In the lab yeast pellets were fed to larvae in a series of trials. One small 50 mg tablet of inactivated yeast was found to kill up to 20 mosquito larvae.

Demonstration of the yeast pellets

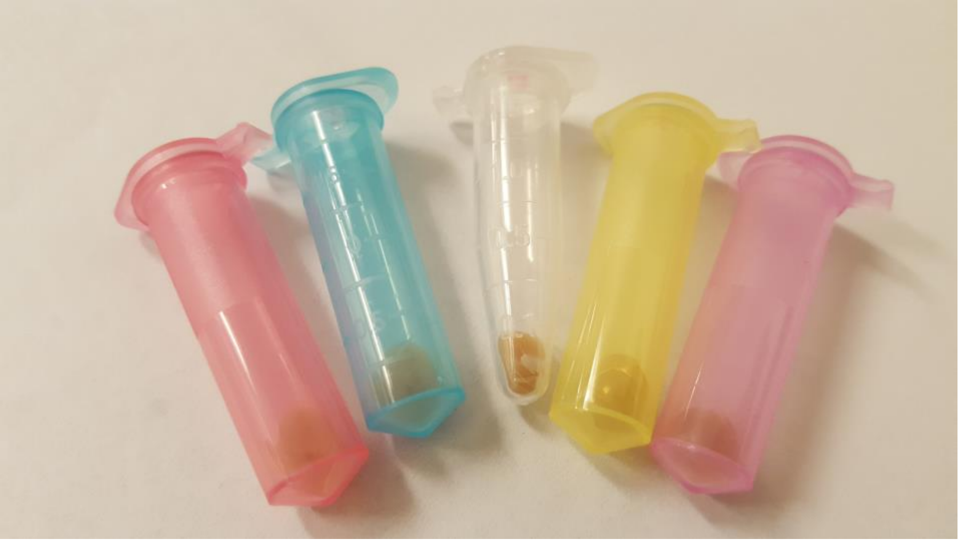

Fig 5. Demo of yeast pellets. Yeast pellets in Eppendorf tubes that we distributed during the engagement

We believe that these lure-and-kill ovitraps represent a safe and effective way to overcome pesticide resistance and concerns for the use of chemicals in the environment.
