# Supplementary material for: Assessment of Trinidad community stakeholder perspectives on the use of yeast interfering RNA-baited ovitraps for biorational control of Aedes mosquitoes
Source: PLoS One. 2021 Jun 29;16(6):e0252997. doi: 10.1371/journal.pone.0252997 (PMC8241094; doi:10.1371/journal.pone.0252997)
Supplement: S6 File — This series of questions was posed by the moderators during the engagement forums. (PDF) [file pone.0252997.s006.pdf]

Larvicidal Ovitrap Community Engagement Forum Study

Script

Interviewer: *We want to thank you very much for participating in our study of new ways to control mosquitos, which breed in standing water. As we move forward, it is very important for us to hear directly from you about your thoughts and feelings pertaining to the larvicides and ovitraps we are studying.*

1. *Ovitraps lure female mosquitos ready to lay eggs, and larvicides prevent mosquito larvae from surviving and developing into adult mosquitoes which can bite and carry disease. Our work on this project combines ovitraps and larvicides.* What is your impression of how well the larvicidal ovitraps we have developed may work to control mosquitoes on your property? What do you think of this approach?

2. Is there anything about the larvicidal ovitraps we described that you particularly like? *We are interested in learning about ways our approach to controlling mosquitos might appeal to users more than alternative approaches.*

3. Is there anything about the larvicidal ovitraps we described that you did not like? *We are interested in learning about ways to improve our larvicidal ovitraps.*

4. When you think about choosing among product options for mosquito control on your property, which factors are most important to you?

5. It the larvicidal ovitraps we described were available for purchase, would you be interested in buying them? If so, what do you think a reasonable price for a monthly supply would be?

6. Is there anything else you would like to tell us about the larvicidal ovitraps we are testing?

Interviewer: *We thank you for participating in this interview. Your input is very important to us.*

Larvicidal Ovitrap Community Engagement Forum Study

Follow Up Question Bank

______ = word or phrase the respondent has already said in the initial answer to an interview question OR word or phrase in the interview question

Could you tell me more about ______?

What do you mean by ______?

Could you explain ______ to me further?

Could you give me some more details about ______?

What other words would you use to describe ______?

Can you give me an example of ______?

What happened after ______?

Interviewer: *We thank you for participating in this interview. Your input is very important to us.*
