# Supplementary material for: Assessment of Trinidad community stakeholder perspectives on the use of yeast interfering RNA-baited ovitraps for biorational control of Aedes mosquitoes
Source: PLoS One. 2021 Jun 29;16(6):e0252997. doi: 10.1371/journal.pone.0252997 (PMC8241094; doi:10.1371/journal.pone.0252997)
Supplement: S7 File — This information sheet was provided to interview participants prior to conducting the interviews. (PDF) [file pone.0252997.s007.pdf]

Study Information Sheet

Larvicidal Ovitrap Trial Participant Feedback Interview Study

You are invited to participate in an interview to collect your thoughts about use of mosquito larvicides and ovitraps in Trinidad, and feedback about the larvicidal ovitraps in this study. The people of Trinidad can contract serious diseases through the bites of mosquitoes, and a research project being conducted here will investigate new ways of preventing mosquitoes from transmitting infections.

This study is being conducted by Dr. Azad Mohammed (The University of the West Indies at St. Augustine, Trinidad and Tobago), as part of a larger research project led by Dr. Molly Duman Scheel of Indiana University School of Medicine-South Bend, in collaboration with Dr. David W. Severson and Nicole Achee of the University of Notre Dame (United States). You are invited to participate in this study because you are an adult resident of Trinidad. If you have any questions about this study, please contact Dr. Molly Duman Scheel at (574) 631-7194 (country code 1) or mscheel@nd.edu. For questions about your rights as a research participant, to discuss problems, complaints, or concerns about a research study, or to obtain information or offer input, contact the IU Human Subjects Office at 317-278-3458.

This study consists of a set of interview questions intended to solicit your feedback about the larvicidal ovitraps we are testing. As our research project pursues new ways of preventing mosquitoes from carrying disease, it is very important for us to consult adults in households where larvicidal ovitraps may be used. The purpose of the study is to collect feedback from adults whose households participated in the larvicidal ovitrap field trials, including relevant observations, thoughts, feelings, and opinions.

Participation in this study is voluntary and you may choose to stop participating in this study at any time.

This study consists of an oral interview, which should take 10-20 minutes to complete, and a written questionnaire to collect some basic information about yourself and your household, which should take 1-3 minutes to complete.

If you agree to participate in this study, you will be asked to speak to us about various aspects of your experience with the field trial of our mosquito larvicidal ovitraps by answering the questions asked by the interviewing researcher. Your spoken responses will be audio recorded. You will also be asked to enter some basic information about yourself and your household on a paper form, which will be collected by the interviewing researcher.

Participating in this study may not provide any direct benefit to you. The knowledge gained from this study will be used as part of a large research project that may result in the development of new mosquito larvicidal ovitraps, ultimately intended to disrupt the transmission of human disease. We do not envision any significant risks related to participation in this study. Privacy with respect to information you will share with us if you participate in this study will be protected by the investigators. Audio recordings will be converted to digital transcripts which will not contain any individually identifying information, and the original recordings of your voice will be deleted. Your name and other information which would allow you to be identified as an individual are not being collected in this study, so your spoken and written responses will not be attributable to you.

Thank you for agreeing to participate in our research. Before you begin, please note that this research is for residents of Trinidad over the age of 18 whose households participated in the larvicidal ovitrap field trial; if you are not a resident of Trinidad your household did not directly participate in the larvicidal ovitrap field trial, and/or you are under the age of 18, please do not participate in this study.
