# Supplementary material for: Assessment of Trinidad community stakeholder perspectives on the use of yeast interfering RNA-baited ovitraps for biorational control of Aedes mosquitoes
Source: PLoS One. 2021 Jun 29;16(6):e0252997. doi: 10.1371/journal.pone.0252997 (PMC8241094; doi:10.1371/journal.pone.0252997)
Supplement: S8 File — This optional demographic sheet was provided to interviewees. (PDF) [file pone.0252997.s008.pdf]

**Household Participant Feedback Study**

## Demographic questions to accompany interview

**Information about your Household:**

| Question | Answer |
|---|---|
| In the past two years, how many times has someone in your household had dengue, Zika, chikungunya, or yellow fever? If you cannot remember exactly, it is alright to estimate. | |
| How many adults 18-59 years of age live in your household? | |
| How many adults 60 years of age or older live in your household? | |
| How many children under the age of 18 live in your household? | |

**Information about You:**

| Question | Answer | | | | | |
|---|---|---|---|---|---|---|
| What is your gender? | *Male* | | | *Female* | | |
| What is your age in years? | *<20* | *20-29* | *30-39* | *40-49* | *50-59* | *>60* |
| Indicate the highest level of formal education you have completed or in which you are presently enrolled: | *Tertiary* | *Secondary* | *Primary* | *None* | | |
| What is your race? | *Afro-Trinidadian*<br>*Indo-Trinidadian*<br>*European Descent* | | | *Chinese Descent*<br>*Mixed Descent*<br>*Other* | | |
