# Supplementary material for: Assessment of Trinidad community stakeholder perspectives on the use of yeast interfering RNA-baited ovitraps for biorational control of Aedes mosquitoes
Source: PLoS One. 2021 Jun 29;16(6):e0252997. doi: 10.1371/journal.pone.0252997 (PMC8241094; doi:10.1371/journal.pone.0252997)
Supplement: S9 File — This series of questions was posed by the interviewer during the interview study. (PDF) [file pone.0252997.s009.pdf]

Larvicide Trial Participant Feedback Study
Script

Interviewer: *We want to thank you very much for participating in our study of new ways to control mosquitos, which breed in standing water. As we move forward, it is very important for us to hear directly from you about your thoughts and feelings pertaining to the larvicides and ovitraps we are studying.*

1. *We would like to know about your experience using the larvicidal ovitraps we are testing.* **How easy or difficult were these products to use?**

2. **What did you notice, if anything, about having these larvicidal ovitraps on your property?** *We would like to hear about anything you observed with your senses, including what you might have seen, smelled, touched, or heard.*

3. *Ovitraps lure female mosquitos ready to lay eggs, and larvicides prevent mosquito larvae from surviving and developing into adult mosquitoes which can bite and carry disease.* **What is your impression of how well the products we are testing functioned as ovitraps (attracting egg-laying females) and larvicides (killing any larvae that developed from the eggs)?**

4. **If you have used any sort of ovitraps in the past, how do the larvicidal ovitraps we are testing compare with others you are familiar with?** *We are interested in hearing about both similarities and differences.*

5. **Is there anything about the larvicidal ovitraps we are testing that you particularly liked?** *We are interested in learning about ways our approach to mosquito control might appeal to users more than other types of approaches.*

6. **Is there anything about the larvicidal ovitraps we are testing that you did not like?** *We are interested in learning about ways to improve our larvicidal ovitraps.*

7. **If the larvicidal ovitraps we are testing were available for purchase, would you buy them? If so, what do you think a reasonable price for a monthly supply would be?**

8. **Is there anything else you would like to tell us about the larvicidal ovitraps we are testing?**

Interviewer: *We thank you for participating in this interview. Your feedback is very important to us.*

Larvicide Trial Participant Feedback Study

Follow Up Question Bank

______ = word or phrase the respondent has already said in the initial answer to an interview question OR word or phrase in the interview question

Could you tell me more about ______?

What do you mean by ______?

Could you explain ______ to me further?

Could you give me some more details about ______?

What other words would you use to describe ______?

Can you give me an example of ______?

What happened after ______?

Interviewer: *We thank you for participating in this interview. Your feedback is very important to us.*
