# Supplementary material for: Assessment of Trinidad community stakeholder perspectives on the use of yeast interfering RNA-baited ovitraps for biorational control of Aedes mosquitoes
Source: PLoS One. 2021 Jun 29;16(6):e0252997. doi: 10.1371/journal.pone.0252997 (PMC8241094; doi:10.1371/journal.pone.0252997)
Supplement: S1 Fig — A summary of the three approaches used for data collection is provided. (PDF) [file pone.0252997.s010.pdf]

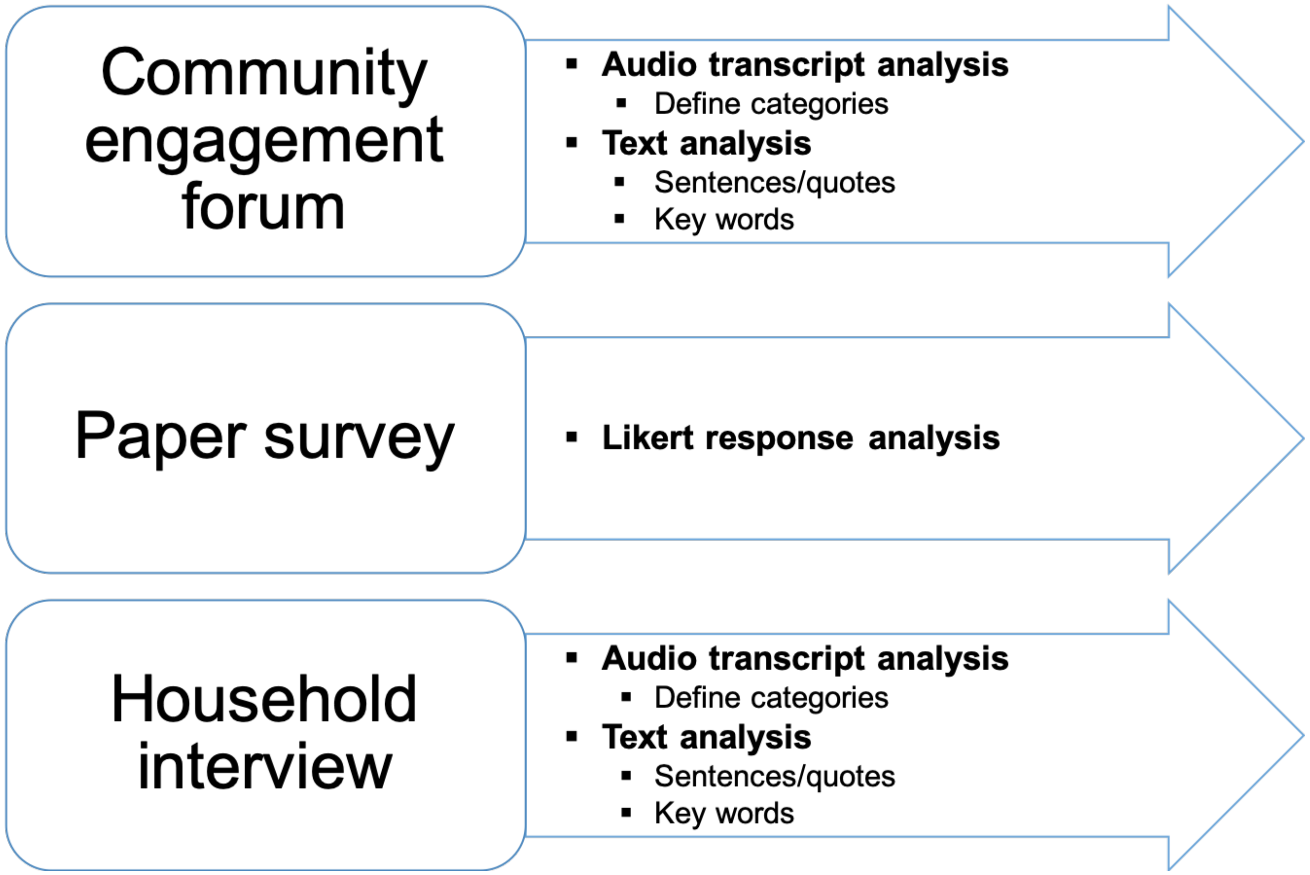
Community engagement forum
Audio transcript analysis
Define categories
Text analysis
Sentences/quotes
Key words
Paper survey
Likert response analysis
Household interview
Audio transcript analysis
Define categories
Text analysis
Sentences/quotes
Key words
