# Supplementary figures and images for: Assessment of Trinidad community stakeholder perspectives on the use of yeast interfering RNA-baited ovitraps for biorational control of Aedes mosquitoes
Source: PLoS One. 2021 Jun 29;16(6):e0252997. doi: 10.1371/journal.pone.0252997 (PMC8241094; doi:10.1371/journal.pone.0252997)

**Age**  
(n=86)

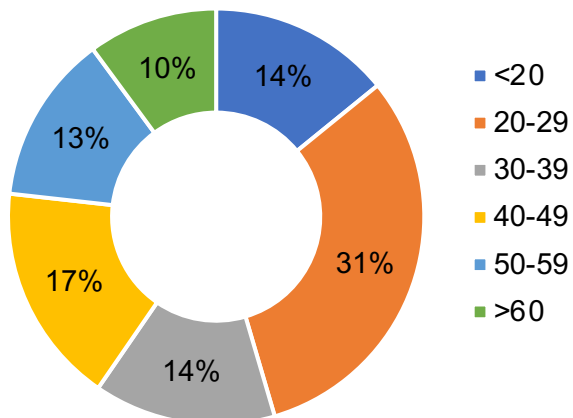

**Gender**  
(n=85)

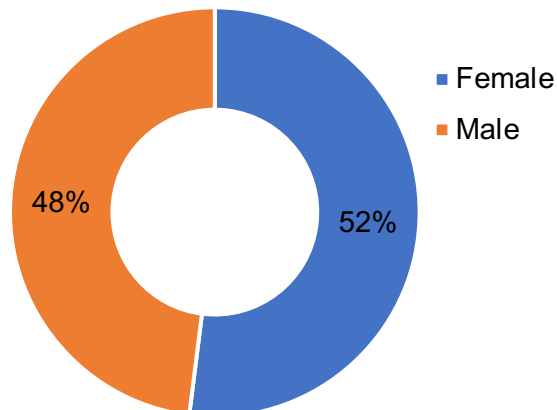

**Education Level**  
(n=86)

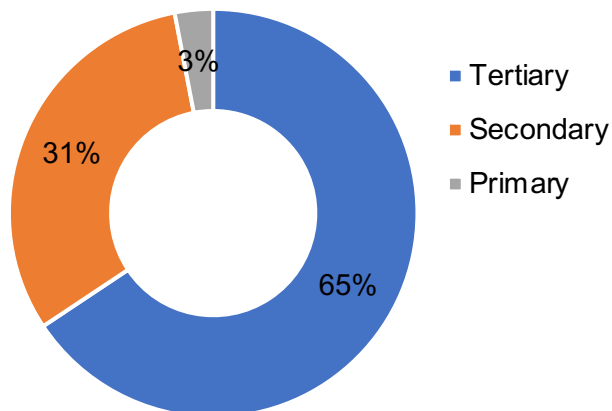

**Race**  
(n=86)

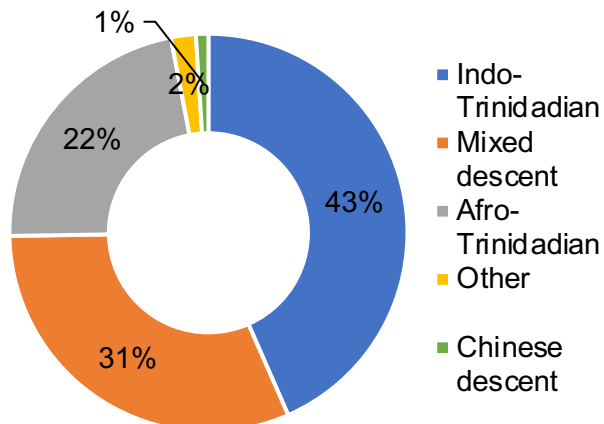

Supplement: S2 Fig — Data for participants’ age, gender, race and education are shown. (PDF) [file pone.0252997.s011.pdf]

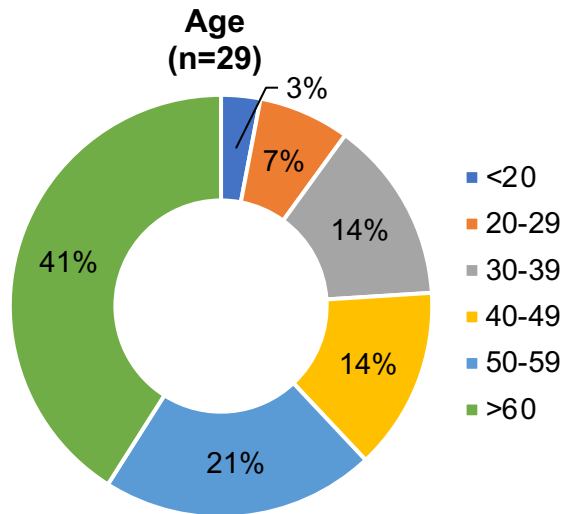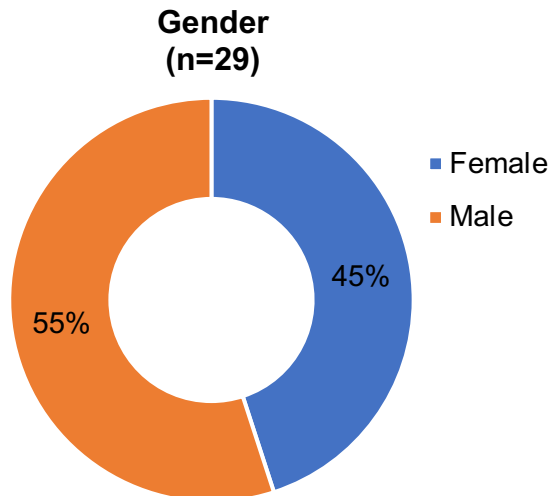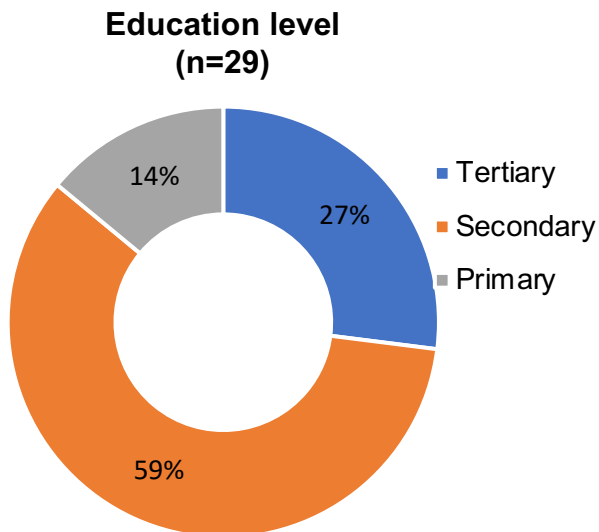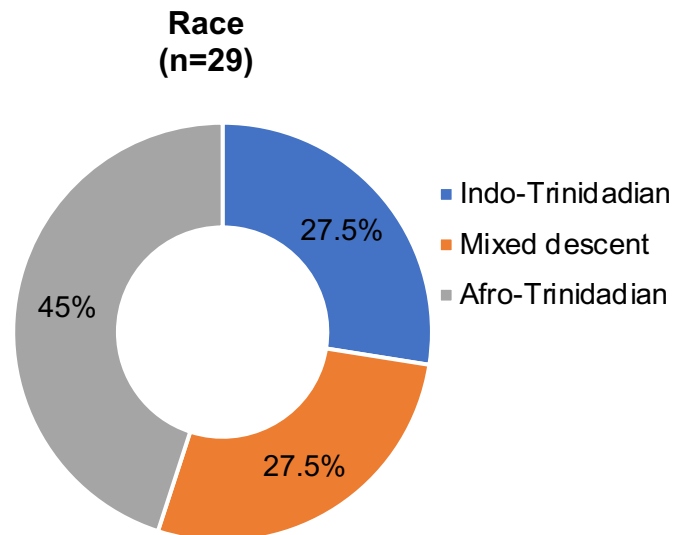

Supplement: S3 Fig — Gender, age, race and education of householder interviewees are shown. (PDF) [file pone.0252997.s012.pdf]
