# Supplementary material for: Assessment of Trinidad community stakeholder perspectives on the use of yeast interfering RNA-baited ovitraps for biorational control of Aedes mosquitoes
Source: PLoS One. 2021 Jun 29;16(6):e0252997. doi: 10.1371/journal.pone.0252997 (PMC8241094; doi:10.1371/journal.pone.0252997)
Supplement: S1 Table — The counts and percentages of the total surveys collected are detailed for major areas across Trinidad. (PDF) [file pone.0252997.s013.pdf]

Location collected	Number collected	% of Total
Central	68	13
Northwest	69	13
East-West-Corridor	162	32
South	59	12
UWI	155	30
Total	513	100
