# Supplementary material for: Assessment of Trinidad community stakeholder perspectives on the use of yeast interfering RNA-baited ovitraps for biorational control of Aedes mosquitoes
Source: PLoS One. 2021 Jun 29;16(6):e0252997. doi: 10.1371/journal.pone.0252997 (PMC8241094; doi:10.1371/journal.pone.0252997)
Supplement: S2 Table — The location, date and number of attendees for each community engagement forum are detailed. (PDF) [file pone.0252997.s014.pdf]

Engagement Forum	Location	Date	# of Attendees
UWI	UWI, Department of Life Sciences Conference Room	9/10/2018	37
St. Augustine-1	Old Tim Road Residential Home	3/11/2019	20
St. Augustine-2	St. Augustine South Community Centre	3/13/2019	14
St. Augustine-3	St Augustine Secondary School	3/21/2019	42
