# Supplementary material for: Assessment of Trinidad community stakeholder perspectives on the use of yeast interfering RNA-baited ovitraps for biorational control of Aedes mosquitoes
Source: PLoS One. 2021 Jun 29;16(6):e0252997. doi: 10.1371/journal.pone.0252997 (PMC8241094; doi:10.1371/journal.pone.0252997)
Supplement: S3 Table — Word count analyses revealed ten commonly repeated words. Commonly repeated words, the number of times that the words appeared (among a total of 5,246 words across the four community engagement forums), and quotes that exemplify the context in which the words were often used are shown. (PDF) [file pone.0252997.s015.pdf]

Theme	Keywords	Count	Percentage	Quotes Representing this Theme
**Awareness**	Disease/s	6	1.3	So, this carries diseases?
	Mosquitoes/other	8	1.8	Adult or pupa?
Total		**14**	**3.1**	
**Containers**	Bucket/s	35	7.7	Is it that it is normal yeast we use at home that could be poured in a bucket or in a small drain?
	Containers/other	9	2.0	You have the barrel full up there with water. Would mosquito go in there?
Total		**44**	**9.7**	
**Current control**	Indoor spraying	13	2.8	I use 'branded insecticidal spray', and I breathing in too much 'branded insecticidal spray' already.
	Outdoor/other	7	1.5	You have to run outside to cover your fishes and whatever animal you have outside there because sometimes they spraying they come unannounced.
Total		**21**	**4.3**	
**Efficacy**	Better/Work/s	13	2.8	In my head I think it will work.
	Effective	9	2.0	How effective would the trap be seeing that the drain will be a competitor?
	Kill/s	7	1.5	Some of them have a high scent that could kill you instantly.
Total		**29**	**6.3**	
**Mosquito biology**	Lifecycle	29	6.3	How does this pellet work and at what stage of the mosquito lifecycle you have to address thereafter?
	Feeding/Behavior	10	2.2	Is there any particular organism, like a particular bug or bee, that feeds on this larva alone?
Total		**39**	**8.5**	
**Operations**	Authorities	18	3.9	Is the government being informed or are they assisting in any way?
	Time/Supply	12	2.6	A month supply. So, you put the yeast in the bucket and the mosquito do not go there?
	Clean/ing	9	2.0	I could spend two minutes, every three to six months to clean a bucket that completely eradicates mosquitoes from my home.
Total		**39**	**8.5**	
**Product design**	Application	35	7.7	How long would the potency last?
	Composition	17	3.7	It has to be self-cleaning or some sort of thing or biodegradable components.
	Usability	13	2.8	Easy to use.
	Function	11	2.4	How often would you need to replace the yeast?
	Appearance	9	2.0	Is it that they [ovitrap] will consistently be the same height and size?
	Smell	8	1.8	So that would prevent the smell you normally get from the chemical.
	Cost	6	1.3	I think it is a really good cost-effective methodology that you all approached.
Total		**99**	**21.7**	
**Safety**	Fauna /Flora	31	6.8	I a little concerned about the food chain along the line…as in the stuff that feed on the mosquitoes, which would be like lizards.
	Humans/Harmful	25	5.5	It could have effects on your system.
	Genetically modified /Natural	13	2.8	I know you mentioned that the yeast is genetically modified so, is it safe to put it in drinking water?
	Threats	11	2.4	I pump water from my pond, does this affect my plants?
	Humans/Exposure	11	2.4	I have two young children so that [approach] would prevent the smell you normally get from the chemical.
Total		**91**	**19.9**	
**Vector control**	Water	31	6.8	It is like a big drain and sometimes the water is not running and the mosquitoes breed in there.
	Breeding sites	27	5.9	How effective would the trap be seeing that the drain will be a competitor?
	Practices	15	3.3	The bad thing is that it is outside range so that we are not really fixing the problem, we are just protecting ourselves.
	Concerns	9	2.0	With chemicals I am concerned about their safety
Total		**82**	**17.9**	
**Keyword Total**		**457**	**100**	
