# Supplementary material for: Assessment of Trinidad community stakeholder perspectives on the use of yeast interfering RNA-baited ovitraps for biorational control of Aedes mosquitoes
Source: PLoS One. 2021 Jun 29;16(6):e0252997. doi: 10.1371/journal.pone.0252997 (PMC8241094; doi:10.1371/journal.pone.0252997)
Supplement: S4 Table — The household number, number of individuals interviewed at each household, interview dates, and number of ovitraps placed at each residence are detailed. (PDF) [file pone.0252997.s016.pdf]

Household Number	Interview date	Number of ovitraps	Number of interviewees
1	8/14/2019	2	1
2	8/14/2019	1	1
3	8/14/2019	3	1
4	8/21/2019	2	1
5	8/21/2019	3	2
6	8/27/2019	4	2
7	8/27/2019	3	1
8	8/07/2019	2	1
9	9/03/2019	1	1
10	9/03/2019	2	1
11	9/03/2019	1	1
12	9/03/2019	2	2
13	9/04/2019 + 10/23/2019	3	2
14	9/04/2019	2	1
15	9/10/2019	1	1
16	9/10/2019	1	1
17	9/18/2019	1	1
18	9/18/2019	1	1
19	9/18/2019	1	1
20	9/04/2019	1	1
21	9/10/2019	2	2
22	10/16/2019	2	2
23	10/23/2019	3	1
**Total**		**44**	**29**
