# Supplementary material for: Assessment of Trinidad community stakeholder perspectives on the use of yeast interfering RNA-baited ovitraps for biorational control of Aedes mosquitoes
Source: PLoS One. 2021 Jun 29;16(6):e0252997. doi: 10.1371/journal.pone.0252997 (PMC8241094; doi:10.1371/journal.pone.0252997)
Supplement: S5 Table — Word count analyses revealed ten themes associated with commonly repeated words. Common words associated with each theme, the number of times that the words appeared (among a total of 13,147 words across 29 individual interviews), and quotes that exemplify each theme are shown. (PDF) [file pone.0252997.s017.pdf]

Theme	Keywords	Count	Percentage	Quote Representing this theme
**Awareness**	Knowledge	11	1.3	I observe the eggs, I see all you put the paper so, I a man like to gain knowledge too.
	Educate/d/ing	8	1.0	This what all of you doing here is also a chance at educating people.
Total		**19**	**2.3**	
**Containers**	Bucket/s	61	7.4	Yeah because sometimes I watch the bucket too and it does pull real eggs in it.
	Other vessels	17	2.1	People keep tires as ways to block driveways like pre-barrier things… What they can do is drill holes around the tire and let the water runoff
**Total**		**78**	**9.5**	
**Current control**	Indoor spray	36	4.3	Yes, it is better; the ability to track the mosquitoes unlike using the spray and other insecticidal things.
	Other control	22	2.7	Throw a little bleach in it. That go kill out whatever bacteria or mosquito or whatever.
	Outdoor spray	17	2.1	The Insect Vector sprayed but this is the first time that I am hearing about the ovitrap.
	Local control	14	1.7	What we does usually do in the drain and them we does like put oil but we does not full up the whole drain in oil so whatever larva it have it will kill them.
	Electronic devices	9	1.1	On an evening I come out with my zapper and I zap all the mosquitoes when they gather by the door there.
Total		**98**	**11.9**	
**Efficacy**	Work/s/ing	44	5.3	It has to be working good because I find I am getting way less mosquito bites in the night because I live downstairs
	Effective/ Decrease	43	5.2	I have an idea what is going on and I am seeing that, you know, you all are catching the eggs so, of course, there is a decrease.
	Check/Difference	23	2.8	It is only five minutes it takes to come, check it out and replace the water and everything
	Kill/s/dead/die/d	13	1.6	And the fogging does raise the mosquito…It killing we more than it is killing them
Total		**123**	**14.8**	
**Mosquito biology**	Life cycle	53	6.4	So, all of yours approach to me is the best approach because all of you are taking it from first-hand. The young ones so, all you are not allowing them to turn into adults.
	Behavior	17	2.1	The different type of mosquitoes and which ones, like how you now say it have the daytime one and it have the night-time one.
Total		**70**	**8.4**	
**Operations**	Come/ing	74	8.9	It is only five minutes it takes to come, check it out and replace the water and everything like that.
	Time/Refill	42	5.1	I do not know how much all you does use one time.
Total		**116**	**14.0**	
**Product Design**	Application	41	4.9	To me, it is an easy thing to maintain.
	Usability/ Appearance	38	4.6	I really do not know what could attract these mosquitoes and trap them besides the bucket
Total		**79**	**9.5**	
**Safety**	Environmental	16	1.9	I have a little puppy in the yard here and if it happen to fall and he lick it, what if it kill him?
	Humans/Exposure	12	1.4	Imagine mosquito leave your whole body and bite you on your nose on top your eye and only he alone he biting so, we does be kind of scared because he is a baby.
	Humans/Harmful	8	1.0	Is it safe to be around humans?
Total		**36**	**4.3**	
**Vector control**	Concerns	66	8.0	Here is a mosquito infestation, right here, that could be because of the drains.
	Water	43	5.2	Everybody need to contain they water, how they storing water and how they having water. So, basically, we have an individual problem.
	Breeding	36	4.3	I do not try to encourage any breeding space for them.
	Methods	48	5.8	We have a big problem, with garbage if we throw a whole bag there and rain fall and mosquito need about that [small amount of] water.
	Practices	17	2.1	I have two covers with spandex material… so those barrels covered all the time, so those little devils cannot get in.
Total		**210**	**25.3**	
**Keyword Total**		**829**	**100**	
